# ALCAT1 Overexpression Affects Supercomplex Formation and Increases ROS in Respiring Mitochondria

**DOI:** 10.1155/2019/9186469

**Published:** 2019-12-06

**Authors:** Bettina Rieger, Adéla Krajčová, Patrick Duwe, Karin B. Busch

**Affiliations:** ^1^Institute of Molecular Cell Biology, Department of Biology, University of Muenster, Muenster, Germany; ^2^Department of Biochemistry, Cell and Molecular Biology, Third Faculty of Medicine, Charles University in Prague, Czech Republic

## Abstract

Cardiolipin (CL) is a multifunctional dimeric phospholipid that physically interacts with electron transport chain complexes I, III, and IV, and ATP synthase (complex V). The enzyme ALCAT1 catalyzes the conversion of cardiolipin by incorporating polyunsaturated fatty acids into cardiolipin. The resulting CL species are said to be more susceptible to oxidative damage. This is thought to negatively affect the interaction of cardiolipin and electron transport chain complexes, leading to increased ROS production and mitochondrial dysfunction. Furthermore, it is discussed that ALCAT1 itself is upregulated due to oxidative stress. Here, we investigated the effects of overexpression of ALCAT1 under different metabolic conditions. ALCAT1 is located at the ER and mitochondria, probably at contact sites. We found that respiration stimulated by galactose supply promoted supercomplex assembly but also led to increased mitochondrial ROS levels. Endogeneous ALCAT1 protein expression levels showed a fairly high variability. Artificially induced ALCAT1 overexpression reduced supercomplex formation, further promoted ROS production, and prevented upregulation of coupled respiration. Taken together, our data suggest that the amount of the CL conversion enzyme ALCAT1 is critical for coupling mitochondrial respiration and metabolic plasticity.

## 1. Introduction

Cardiolipin (CL) is a unique phospholipid that was first identified in 1947 in beef heart [1]. It is also known as the mitochondrial signature lipid [2–4]. Approximately 75% of the CL content in mitochondria is present in the inner mitochondrial membrane (IM), where its biosynthesis takes place [5, 6]. CL is important for mitochondrial function and activity and influences amongst others the electron transport chain (ETC). Therefore, it is not surprising that CL alterations or CL depletion are part of many pathologies. For instance, mutations in the TAZ1 gene, a protein that is important for the final acyl chain composition of CL, lead to an X-linked disease, called Barth syndrome. The disease is characterized by skeletal and cardiac myopathies and cyclic neutropenia, whereas heart deficiency and opportunistic infections are the main reasons of mortality [2, 7]. CL is a negatively charged phospholipid dimer that consists of two phosphatidic acid molecules connected through glycerol. The fact that each CL molecule has four acyl chains differentiate it from all other phospholipids [8, 9].

CL is a multifunctional phospholipid that, under non-pathological conditions, is suggested to participate in different mitochondrial mechanisms such as apoptotic cell death signaling, oxidative phosphorylation (OXPHOS), and fusion and fission events [10, 11]. It interacts with intermembrane space (IMS) or membrane bound proteins such as the electron transport chain (ETC) complexes as well as with soluble proteins, e.g., the phosphotransferase of the IMS, NDPK-D (nucleoside-diphosphate kinase-D), and MtCK (mitochondrial creatine kinase) [11, 12]. The interaction of CL with complex I (CI), III (CIII), and IV (CIV) of the ETC is suggested to support their assembly to respiratory supercomplexes (SCs), which are discussed to favor lower ROS production [13, 14]. The lipid composition of the IM may be important for SC assembly [13]. Studies of SC arrangement propose binding sites of loosely and tightly bound CL [15]. CL was shown to be tightly bound to CI and be necessary for electron transport by this complex [16, 17]. Oxidation of CL and a decreased CL level resulted in less SC formation [15, 18]. Deregulated ETC is seen as the major source of ROS [19–21]. CL also interacts with complex V, the ATP synthase. CL is suggested to promote ATP synthase dimerization, which is relevant for the cristae architecture [22, 23]. Deficiency of CL resulted in an increased level of monomeric ATP synthase in Drosophila [22].

The definite acyl chain composition of one CL molecule is generated in a postsynthetic modification step, called remodeling [9]. Remodeling increases the amount of unsaturated acyl chains in CL [9, 24] and is part of the CL maturation process [25] involving three enzymes: tafazzin (TAZ), monolysocardiolipin acyltransferase 1 (MLCLAT1), and acyl-CoA:lysocardiolipin acyltransferase 1 (ALCAT1) [8, 26]. It is suggested that the remodeling of CL by TAZ is important for sustaining the normal acyl chain composition. ALCAT1 is an Acyl-CoA:lyso-CL acyltransferase and an isoform of MLCLAT1 [8]. CL species in cells overexpressing ALCAT1 had an increased level of polyunsaturated fatty acids, especially DHA (C22:6n3), and a significantly reduced amount of C16-C18 fatty acids [27].

Here, we set out to investigate whether ALCAT1 levels are variable under different metabolic conditions. Moreover, we wanted to determine the effect of artificially overexpressed ALCAT1 under glycolytic and respiratory galactose conditions. We found no significant differences in ALCAT levels under different respiratory conditions due to the high variation in endogenous ALCAT1 levels. ALCAT1 was localized at the ER and mitochondria, probably contact sites. Artificially elevated ALCAT1 levels hampered supercomplex formation and promoted ROS production.

## 2. Materials and Methods

### 2.1. Cell Culture

HeLa WT cells were cultivated in Minimal Essential Medium Earle's Salt (5.6 mM glucose+L-glutamine) supplemented with 10% FCS (fetal calf serum), 1% HEPES, 1% NEAA (nonessential amino acids), and 1% penicillin/streptomycin at 37°C and 5% CO_2_. The cells were split when they were 80-100% confluent using trypsin/EDTA for cell detachment. For cell respiration assays, cells were cultivated in XF Base Medium Minimal DMEM containing 10 mM D-galactose (Seahorse Bioscience, wo D-Glc, wo L-glutamine, phenol red, wo NaHCO_3_) supplemented with 10% FCS, 1% HEPES, 1% NEAA (nonessential amino acids), 1% penicillin/streptomycin, 2% alanyl-L-glutamine, and 2,875% mL NaHCO_3_. HeLa cells were purchased from the Leibniz Institute DSMZ-German Collection of Microorganisms and Cell Cultures and transiently transfected by the calcium transfection method if required. The transfected cells were used 48-72 h after transfection.

The following plasmids were used for transfection: pSems-ALCAT1-2A-GFP-NLS, pSems-ALCAT1-HA, and pSems-ALCAT1-mCherry. Sequences of the according plasmids are shown in the supplementary material. pSems-ALCAT1-2A-GFP-NLS results in the expression of untagged ALCAT1 in parallel with a nuclear-targeted GFP as reporter for successful transfection. Moreover, we used the plasmids CoxVIIIa-sEcGFP and CoxIV-sEcGFP [28].

### 2.2. Seahorse XF Cell Mito Stress Test Kit

The Seahorse XF Cell Mito Stress Test Kit enables metabolic profiling of mitochondria measuring the oxygen consumption rate (OCR) and extracellular acidification rate (ECAR). Sequential injection of inhibitors and uncouplers of the OXPHOS (oligomycin, FCCP, and rotenone/antimycin A) is used to determine basal respiration, proton leak, ATP production, and nonmitochondrial respiration (Supplementary Fig. [Supplementary-material supplementary-material-1]). On the day prior to the measurement, ~30,000 cells per well were seeded in the Seahorse XF Cell Culture Microplate and incubated at 37°C and 5% CO_2_. 180 *μ*L XF Calibrant was added to each well of the calibration hydrate sensor cartridge and incubated at 37°C and 0% CO_2_. Next day, the cells were washed with PBS and incubated in an assay medium for 1 h at 37°C and 0% CO_2_. The inhibitor stock solutions were prepared and mixed with an XF Base medium (5.6 mM glucose or 10 mM galactose, 4 mM glutamine, pH 7.4) to get the constant compound concentrations: 1 *μ*M oligomycin, 1 *μ*M FCCP, and 0.5 *μ*M rotenone/antimycin A. The inhibitor solutions were loaded into the ports of the hydrate sensor cartridge (A: Oligomycin, B: FCCP, and C: rotenone/antimycin A). After calibration of the Seahorse with the hydrate sensor cartridge, basal and ATP synthesis-linked respiration, proton leak, maximal respiration, and nonmitochondrial respiration were measured for 18 min each. The results were analyzed with the Seahorse XF Stress Test Report Generator (XF Cell Mito Stress Test Kit User Guide).

### 2.3. Immunoblotting

Cells, grown in a glucose or galactose medium for 48 h, were harvested from T25-flask dishes in SDS sample buffer by using a cell scraper and transferred to a 1.5 mL reaction tube. After vortexing, the cell lysate was incubated on ice for 10 min and then boiled at 95°C for 5 min. The SDS samples were stored at -20°C. The SDS-PAGE run was performed at 60 V. When the samples reached the separation gel, the voltage was increased to 100-150 V. PageRuler Prestained Protein Ladder (product #26619, ThermoFisher Scientific) was used as a marker. The proteins were transferred to a PVDF membrane, and the blotting was performed in a semidry manner at 100 mA for 2 h. After blotting, the membrane was incubated with Ponceau S for 10 min to control the protein transfer. Ponceau S was washed away with MilliQ, and the membrane was blocked with 10 mL of 10% skimmed milk/TBST (20 mM Tris; 0.137 M NaCl, 0.1% Tween 20) for 1 h to prevent unspecific binding of the primary antibody. The primary antibody was diluted in 1% skimmed milk/TBST and was added to the membrane overnight at 4°C. Then, the membrane was washed three times with TBST for 15 min and incubated for 1 h with the secondary antibody, also diluted in 1% skimmed milk in TBST. Again, the membrane was washed three times with TBST for 15 min. Finally, SuperSignal West Pico Chemiluminescent Substrate 1 : 1 (ThermoFisher Scientific) were added to the membrane and the fluorescent signal was detected with the ChemiDoc MP Imaging System (BIO-RAD). The primary antibodies that were used for the protein expression analysis are listed in Supplementary [Supplementary-material supplementary-material-1]. The peroxidase-conjugated AffiniPure Goat Anti-Rabbit IgG (H+L) (Jackson ImmunoResearch, Dianova) was used as a secondary antibody at a final dilution of 1 : 2000.

Images of Western Blot results were processed with Adobe Photoshop, and quantitative analysis of protein levels was executed with ImageJ™ (MacBiophotonics). The mean and integrated density grey values of the protein of interest were normalized with VDAC (voltage-dependent anion channel), a mitochondrial membrane protein that is correlated with mitochondrial mass. For two-sample, two-sided *t*-test statistical analysis, Origin (OriginPro 2016, OriginLab) was used.

### 2.4. Microscopy

For microscopy, the cells were seeded on coverslips in 3 cm dishes (70% confluency) in a medium with glucose or galactose as sugar source for 48 h. For imaging, the coverslips were mounted in a cell chamber supplied with 1 mL medium. The cells were imaged using an inverted confocal microscope (TCS SP8 SMD, Leica) equipped with a 63x water objective (N.A. 1.2) and a Time-Correlated Single Photon Counting (TCSPC) device. Living cells were imaged at 37°C supplied with 5% CO_2_. Recording of the fluorescence lifetime of excited CoxVIIIa-sEcGFP and CoxIV-sEcGFP was performed by TCSPC. HyD's with GaASP photocathodes were used as detectors for dual color recording and FLIM (fluorescence lifetime imaging microscopy). For FLIM, emission was restricted to 525/50 nm (ex. 488 nm, 20 MHz). The acquisition was performed until at least 1,000 photons in the brightest pixel were reached. Data analysis was performed with SymphoTime software (64 bit) and biexponential fitting of the fluorescence decay curves (substracting the IRF) from ROIs of the whole mitochondrial network of a cell. From biexponential fits, the average lifetime *τ*_amp_ was calculated. For standard microscopy, HeLa WT cells were transiently transfected with ALCAT1-2A-GFP-NLS plasmid DNA, except for localization of ALCAT1. For FLIM, HeLa WT cells were transiently transfected with ALCAT1-mCherry and either CoxVIIIa-sEcGFP or CoxIV-sEcGFP.

### 2.5. Mitochondrial Membrane Potential, Mitochondrial Footprint, and ROS Determination

To determine the morphology under different nutrition conditions, HeLa WT and ALCAT1-2A-GFP-NLS expressing cells were cultivated in a glucose and galactose medium for 48 h and stained with 100 nM MitoTracker Red under normal growth conditions (37°C, 5% CO_2_) for 30 min. Then, the cells were washed two times with PBS and one-time with a medium to remove the dye. The fluorescence of ~45 cells was examined with the fluorescence microscope (TCS SP8, Leica) with a 63x water-immersion objective. The excitation source was a pulsed supercontinuum white light laser (Leica Microsystems, at 80 MHz). The emission of nuclear-targeted GFP was set to 510-540 nm (ex. 488 nm), and the emission of MitoTracker Red was colleced between 600-700 nm (ex. 561 nm), recorded by hybrid detectors. Finally, the mitochondrial morphology (mean branch length and mitochondrial footprint) was analyzed with the ImageJ™ plugin "MiNA" (Valente et al., 2017). For determination of ROS, MitoTracker Red was used. MitoTracker Red is a reduced, nonfluorescent version of MitoTracker Red (M-7512, ThermoFisher) that fluoresces upon oxidation. Z-stacks of 7 slices (step size of 360 nm) of the MitoTracker Red stained cells were recorded with the same settings as described above. The fluorescence intensity was analyzed with ImageJ (MacBiophotonics). To exclude the background intensity, the Otsu mask was used for thresholding. A higher grey value corresponds to a higher ROS level, respectively mitochondrial membrane potential. For determination of the mitochondrial membrane potential, the cells were stained with 7 nM TMRE instead of 100 nM MitoTracker Red CM-H2XRos.

### 2.6. Localization of ALCAT1

HeLa WT cells were cotransfected with ALCAT1-mCherry plasmid and CoxIV-sEcGFP plasmid DNA. The antibody anti-KDEL from Santa Cruz Biotechnology (10C3; 1 : 50) served as a marker of the ER. As secondary antibody, Goat anti-mouse IgG H&L (Alexa Fluor® 647; 1 : 500) was used. Briefly, antibody staining was performed after cell fixation with 4% paraformaldehyde, permeabilization with 0.1% Triton-X, blocking with 2% BSA, and washing with 0.01% Tween-20, all in PBS. The cells were examined with the fluorescent microscope (TCS SP8, Leica mCherry: ex. 561 nm, em. 585-605 nm; mEGFP: ex. 488 nm, em. 510-530 nm; Alexa 647: ex. 633 nm, em. 720-800 nm). Alternatively, HeLa WT cells were cotransfected with ALCAT1-mCherry plasmid and CoxIV-sEcGFP and stained with LysoTracker blue™ (LysoTracker blue™: ex. 405 nm, em. 420-460 nm; mCherry: ex. 580 nm, em. 610-630 nm; mEGFP: ex. 488 nm, em. 500-535 nm). Scatterplots with linear regression as well as Pearson's and Manders' coefficients were determined with the ImageJ™ plugin “colocalization threshold.”

### 2.7. Statistics

For the comparison of samples with the same sample size, we used ANOVA with post hoc Tukey test; for the comparison of samples with unequal sample size, we used ANOVA with post hoc Scheffe or Fisher LSD for statistics analysis. The box and whisker plots show the 25^th^ to 75^th^ % percentile in the box. The square is the mean; the horizontal line in the box is the respective median of the cohort. Manders and Pearson coefficients were calculated to characterize the degree of overlap of ALCAT1 signal with mitochondria and lysosomes, respectively.

## 3. Results

### 3.1. ALCAT1 Is Localized at the ER and Mitochondria

**Figure 1:**
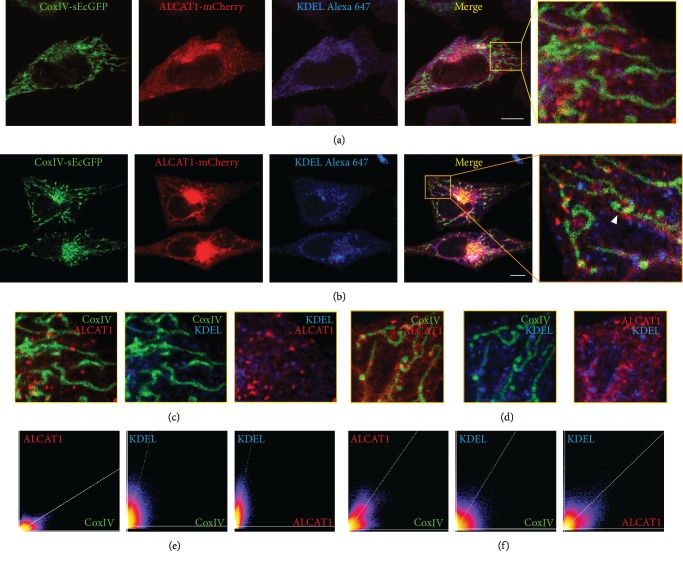
Overexpressed ALCAT1 is localized in the ER and mitochondria. Fluorescence microscopic analysis of transiently transfected cells expressing CoxIV-sEcGFP, ALCAT1-mCherry stained with an antibody against ER marker KDEL. (a) Low ALCAT1-mCherry expression. (b) High ALCAT1-mCherry expression. Arrowheads in zoom image are pointing to ALCAT1-mCherry localized between ER and mitochondria, in other words MAMs. (c) Merged two-color images of ROIs shown in (a). (d) Merged two-color zoom images of ROI shown in (b). (e, f) Colocalization analysis showing scatterplots after setting thresholds in both color channels. (e) Colocalization analysis of the region shown in (a+c), (f) Colocalization analysis of the area shown in (b+d). Upper left quadrant = Ch2 above threshold; Ch1 below threshold; lower left quadrant = Ch1 below threshold; Ch2 below threshold; lower right quadrant = Ch1 above threshold; Ch2 below threshold; upper right quadrant = Ch1 above threshold; Ch2 above threshold; with linear regression; channel 1 (Ch1) = *x*-axis, channel 2 (Ch2) = *y*-axis. Thresholds for scatterplots were automatically determined for each channel (ImageJ plugin “colocalization threshold”), while presented images were manually processed with ImageJ to enhance the contrast. Scale bars: 10 *μ*M (a, b).

It was reported that FLAG-tagged ALCAT1 is localized at the ER [8], while another study reported that ALCAT1 is predominantly localized in the mitochondrial-associated membrane [27]. To test for colocalization between ALCAT1, ER, and mitochondria, HeLa cells were transfected with CoxIV-sEcGFP for mitochondrial staining and ALCAT1-mCherry. To visualize the ER, we performed immune-staining against KDEL, an ER-localized protein, in addition. Alexa Fluor® 647 was used as secondary antibody. We performed triple color analysis in cells with moderate (Figure 1(a)) and high (Figure 1(b)) ALCAT1 expression. Interestingly, we did not only find high overlap between ALCAT1 and KDEL but also quite high overlap between ALCAT1 and CoxIV-sEcGFP, demonstrated by the 2D histograms (scatterplots), where the two intensity values for each pixel (voxel) are plotted against each other. Correlation is visualized by the presence of a cloud of information in the middle of the scatterplot, fitted with a linear regression. The brighter the color, the higher the correlation of intensity values for the two channels (Figures 1(c) and 1(d)). This data clearly shows here that ALCAT1 is not only localized in the ER (colocalization can be seen in Figure 1(f)) but also in mitochondria. However, in order to reveal whether this overlap indeed indicates ER-mitochondria contact sites, further studies using superresolution microscopy are required.

A significant proportion of the overexpressed ALCAT1-mCherry in transfected HeLa cells was found in point-like structures near the nucleus resembling lysosomes (Figure 1). Lysosomal localization of overexpressed proteins is not unusual. To check whether part of ALCAT1 was localized in lysosomes, we conducted three-color fluorescence colocalization microscopy with genetically coded fluorescent and lysosomal markers in cells under glucose conditions. Images were taken of cells expressing ALCAT1-mCherry and CoxIV-sEcGFP and stained with LysoTracker blue™ (Supplementary Fig. [Supplementary-material supplementary-material-1]). Indeed, a significant amount of ALCAT1 is found in lysosomes. The quantitative colocalization analysis showed that the fraction of ALCAT1 colocalizing with lysosomes was higher than the colocalization of ALCAT1 and mitochondria (Supplementary Fig. [Supplementary-material supplementary-material-1]-[Supplementary-material supplementary-material-1]). Together, these data suggest that artificially induced ALCAT1 is associated with different cell organelles including lysosomes. Whether this reflects the natural distribution of ALCAT1 has to be revealed in future studies when suitable ALCAT1 antibodies for immune-staining are available.

### 3.2. Artificially Induced ALCAT1 Is Moderately Overexpressed and Represses Endogenous ALCAT1

**Figure 2:**
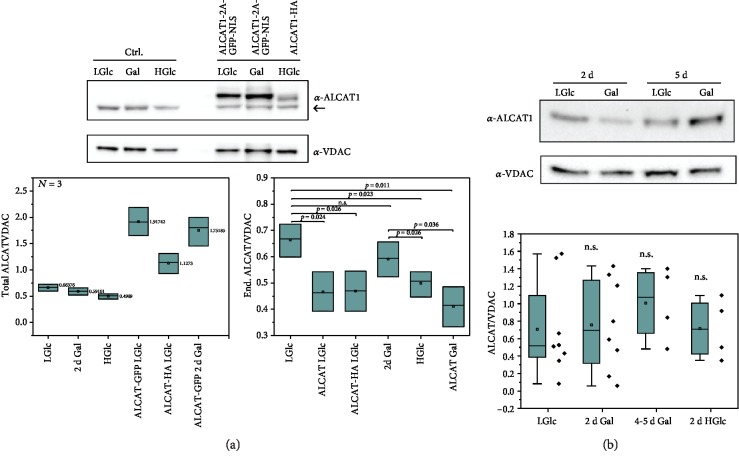
Artificially induced and endogenous ALCAT1 levels. (a) Determination of relative ALCAT1 levels in HeLa cells supplied with different sugars and in cells transiently transfected with ALCAT1-EGFP and ALCAT1-HA-tag, respectively, 48 h after transfection. The transfection efficiency usually was ~60%; the data shown is not corrected for this. The upper band shows the tagged ALCAT1 version, the lower band the endogenous ALCAT1. VDAC, a mitochondrial protein, was used as loading control. For the quantification, ALCAT1 was normalized on the VDAC level. Total ALCAT1 and endogenous ALCAT levels were determined. A-GFP: ALCAT1-2A-GFP-NSL, A-HA: ALCAT1-HA-tag. *N* = 3 biological replicates. Left graph: total ALCAT levels, mean values are indicated. Right graph: only endogenous ALCAT levels are shown. Box and whisker plots. Statistics: ANOVA with post hoc Tukey. (b) Influence of metabolic conditions on endogenous ALCAT1 levels. The quantification includes values from (a). *N* = 8 technical replicates from *n* > 2 biological preparation for ALCAT in low glucose (LGlc; 5.6 mM), *N* = 8/*n* = 3 for galactose (Gal) 2 d; *N* = 4/*n* = 3 for galactose 4-5 d; *N* = /*n* = 2 for high glucose (HGlc, 25 mM). VDAC was used as a loading control. Statistics: SD are shown, ANOVA with post hoc Fisher LSD test. n.s.: no significant differences of the mean values.

**Figure 3:**
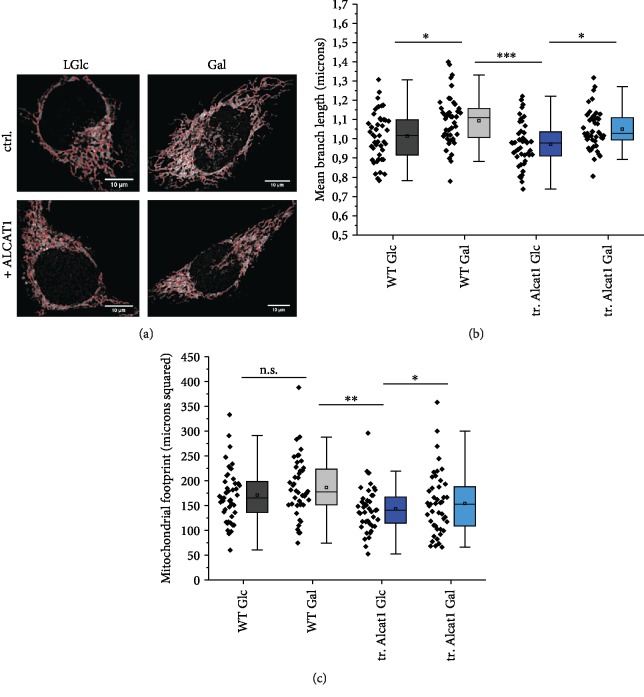
Analysis of mitochondrial morphology in ALCAT1 overexpressing cells. Transiently transfected ALCAT1-2A-GFP-NLS (tr. ALCAT1) and HeLa WT cells were supplied with 5.6 mM glucose and 10 mM galactose, respectively, for 48 h. Quantitative analysis of mitochondrial network morphology by the ImageJ™ plugin MiNA. MitoTracker Red stained mitochondria were analyzed. Morphological data are extracted from the MiNA-processed (= contrast enhanced) images. (a) MiNa-processed images (mitochondrial skeleton in red) of HeLa cells after MitoTracker Red staining of HeLa WT and ALCAT1 overexpressing cells in Glc and Gal (scale bars: 10 *μ*m). (b) Quantitative MiNA analysis of mean branch length of mitochondria. (c) Quantitative MiNA analysis of mitochondrial footprint. The footprint is simply the sum of positive pixels per cell and represents the mitochondrial area. *N* = 2 biological replicates. Each data point represents data from one cell. Statistics: data was analyzed by One-Way ANOVA with post hoc Fisher LSD (^∗∗∗^*p* < 0,001; ^∗∗^*p* < 0.01; and ^∗^*p* < 0.05), SD are shown.

Cells transiently expressing tagged ALCAT1 had in average a 2-3~threefold higher level of total ALCAT1 than the control (Figure 2(a)). This in accordance to what was observed earlier in a different cell line, where the mRNA levels increased threefold [29]. Interestingly, artificially induced ALCAT1 overexpression resulted in a significant decrease of the endogenous ALCAT1 levels (Figure 3(a)). At this stage, we cannot rule out a feedback mechanism that suppresses the transcription of endogenous ALCAT1. The same had already been shown for ALCAT1 overexpressing COS7 cells, but not commented on [8].

### 3.3. Natural ALCAT1 Levels Vary

Natural ALCAT1 levels increase under pathological conditions as in diabetes, obesity, and cardiomyopathy [8, 27]. We asked whether ALCAT1 levels also would change under nonpathological conditions. The activity of mitochondria changes under different metabolic conditions [30], including a remodeling of the OXPHOS system. It can be assumed that this also concerns cardiolipin and its remodeling. Here, we examined whether metabolic changes induced by a change of the sugar source had an effect on ALCAT1 levels. We found no significant changes in ALCAT1 levels when the cells were supplied with galactose or high glucose levels instead of glucose as sugar intake (Figure 2(b)). However, we found a high variation of the endogenous ALCAT1 content under all conditions.

### 3.4. Effects of ALCAT1 on Mitochondrial Morphology

To analyze whether different ALCAT1 levels would affect mitochondrial morphology, we transiently transfected HeLa WT cells with ALCAT1-2A-GFP-NLS and stained the cells with MitoTracker Red™ (Figure 3(a)). The mitochondrial area per cell was determined using an automatic analysis tool (ImageJ™ plugin MiNA). Morphological data were extracted from the binary image that was generated by applying an automatic threshold. A copy of the binary image was overlaid to the original image. From the morphological skeleton, the mean and standard deviation of the branch lengths in each network were determined. The footprint was calculated as the sum of positive pixels (of the binary image) per cell and represents the mitochondrial area. Cells supplied with galactose showed a higher branch length compared to glucose-fed cells (*p* ≤ 0.05). Cells transiently expressing ALCAT1 also had a higher branch length in galactose than in glucose (*p* ≤ 0.05) as seen in Figure 3(b). In glucose, ALCAT1 overexpressing cells did not display a significant change in mitochondrial branch length compared to control cells. No difference in mitochondrial area (footprint) was found between galactose and glucose supplied cells. However, cells that were artificially overexpressing ALCAT1 had a significant reduced mitochondrial area (in *μ*m^2^) compared to control cells (*p* ≤ 0.01) in glucose and ALCAT1 overexpressing cells (*p* ≤ 0.05) in galactose (Figure 3(c)). In sum, we found no relation between ALCAT1 overexpression and mitochondrial morphology under the same metabolic conditions.

### 3.5. Effects of ALCAT1 on Mitochondrial Bioenergetics

**Figure 4:**
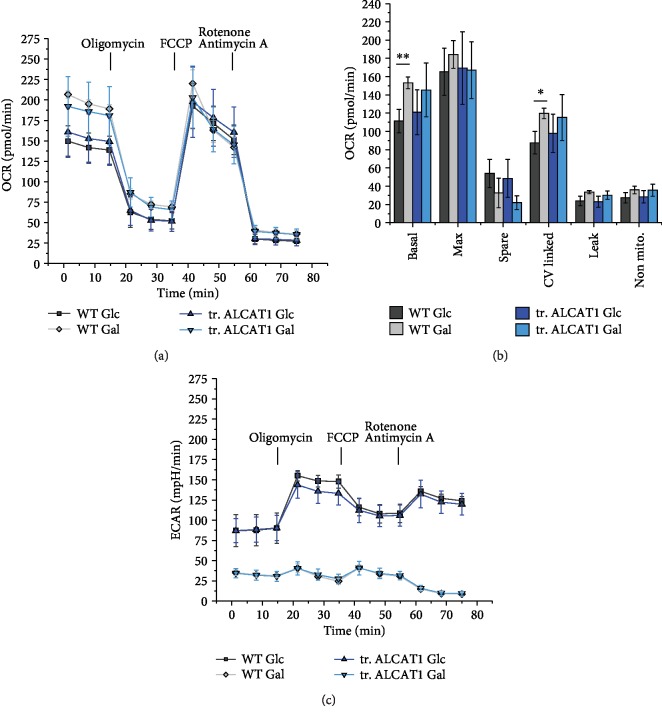
Effects of elevated ALCAT1 levels on mitochondrial bioenergetics. Automatic flux analysis of oxygen consumption rate (OCR) and extracellular acidification rate (ECAR) of HeLa WT supplied with glucose and galactose for 48 h and ALCAT1 overexpressing cells under the same conditions. (a) Oxygen consumption rate (OCR) of HeLa WT and transiently transfected ALCAT1 overexpressing cells. OCRs (and ECAR) were analyzed with the Seahorse XF Cell Mito Stress Test Kit by sequential injection of oligomycin (2 *μ*M), FCCP (1 *μ*M), and rotenone/antimycin A (0.5 *μ*M). The cells were supplied with 5.6 mM glucose or 10 mM galactose for 48 h before measurement. *N* = 5 replicates with 30 wells for each condition. (b) Basal and maximal respiration, spare respiratory capacity, CV-linked respiration, proton leak, and nonmitochondrial respiration determined from OCRs shown in (a). One Way ANOVA with post hoc Scheffe (^∗∗∗^*p* < 0,001; ^∗∗^*p* < 0.01; and ^∗^*p* < 0.05). (c) Corresponding extracellular acidification rates (ECAR).

To analyze possible changes in mitochondrial respiration and ATP synthesis (oxidative phosphorylation, OXPHOS) with respect to ALCAT1 overexpression, we determined the mitochondrial oxygen consumption rate (OCR) and in parallel the extracellular acidification rate (ECAR) with an automatic flux analyzer (Seahorse/Agilent XF 96). We used the stress test as described in Materials and Methods with subsequent addition of the ATP synthase inhibitor oligomycin, the uncoupler carbonylcyanid-p-trifluoromethoxyphenylhydrazone (FCCP), and complex I and III inhibitors rotenone/antimycin A (Supplementary [Supplementary-material supplementary-material-1]). In addition, we compared the OCR and ECAR of cells cultured in glucose or galactose for 48 h. HeLa wild-type (WT) cells showed increased basal and ATP synthesis- (CV-) linked OCR in galactose (Figures 4(a) and 4(b)). With galactose as sugar supply, cells are forced to rely on ATP synthesis by oxidative phosphorylation (OXPHOS) as described earlier [30], shown here as a significant increase in basal- and CV-linked respiration. ALCAT1 overexpression prevented the galactose-induced increase in respiration.

**Figure 5:**
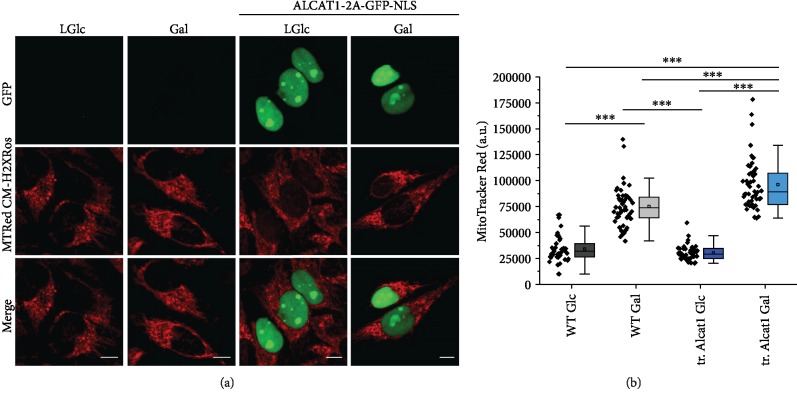
Mitochondrial ROS levels in ALCAT1 overexpressing cells under different nutrition conditions. (a) Dual color images showing ROS formation in cells with normal ALCAT1 levels and cells with artificially elevated ALCAT1 levels (tr. ALCAT1: transiently transfected cells expressing ALCAT1-2A-GFP-NLS). The transfected cells were identified by their green nuclei (GFP::NLS). Cells were supplied with different sugars as indicated before for 48 h. After staining with MitoTracker Red, cells were analyzed with a fluorescence microscope (MitoTracker Red: *λ*_ex._ 561 nm, *λ*_em._ 600-700 nm, GFP: *λ*_ex._ 488 nm, *λ*_em._ 510-540 nm). (b) Quantitative analysis of ROS formation as MitoTracker Red fluorescence intensity signal (arbitrary units, a.u.). Results are presented as box plots with the measured fluorescence intensities of ~45 measured cells. Data was analyzed by One-Way ANOVA with post hoc Scheffe (^∗∗∗^*p* < 0.001; ^∗∗^*p* < 0.01; and ^∗^*p* < 0.05), SD are shown.

Next, the effects of ALCAT1 overexpression on glycolysis are presented. Both HeLa control and ALCAT1 overexpressing cells showed a higher extracellular acidification rate (ECAR) in glucose supply than in galactose, which is a measure of increased glycolysis (Figure 5(c)). This in turn means that the stimulation of respiration evoked by galactose is at the expense of glycolysis. ALCAT1 overexpression had no significant effect on the galactose-induced decrease of glycolysis. Together, these data suggest that high ALCAT1 levels interfere with the OXPHOS stimulation caused by a switch from glucose to galactose, but have no effect on glycolysis rates.

### 3.6. Mitochondrial ROS Levels increase under Elevated ALCAT1 Levels

In isolated mitochondria, the reactive oxygen species (ROS) H_2_O_2_ increased in ALCAT1 overexpressing cells [29]. However, isolated mitochondria tend to lose their ROS detoxifying system which might result in higher ROS levels [31]. We here determined what effects the increased respiration would have on reactive oxygen species (ROS) levels in intact cells with normal and elevated ALCAT1 levels. Glucose and galactose supplied HeLa control and ALCAT1 overexpressing cells were stained with MitoTracker Red, a ROS indicator, to study the effect of ALCAT1 on ROS production under different nutrition conditions. The ROS level was significantly increased in both wild-type and ALCAT1 overexpressing cells supplied with galactose (respiratory conditions) (Figure 5). Only in galactose, ALCAT1 overexpressing cells showed higher ROS formation than cells with normal ALCAT1 levels. The mitochondrial membrane potential was slightly increased in ALCAT1 overexpressing cells under both nutrition conditions compared to HeLa control in glucose (Supplementary Fig. [Supplementary-material supplementary-material-1]). Together, these data show that elevated ALCAT1 levels under respiratory conditions are correlated with increased ROS levels also *in cellulo*.

### 3.7. Effects of ALCAT1 on Respiratory Supercomplex Formation

**Figure 6:**
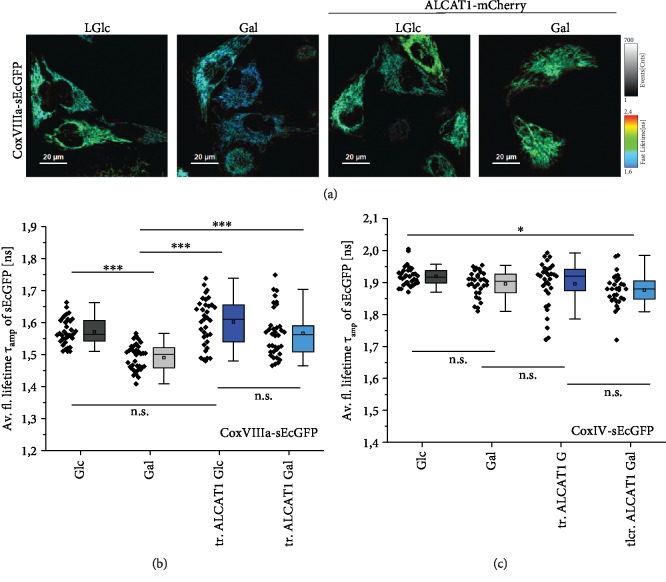
ALCAT1 hampers SC formation under stimulated OXPHOS conditions. (a) Fluorescence lifetime images of sEcGFP at subunit CoxVIIIa. This construct is buried in an existing respiratory supercomplex and has a reduced fluorescence lifetime *τ*. As a control, subunit CoxIV was labeled with sEcGFP, which does not respond to SC formation by decreased lifetime [28]. Cells were transfected with ALCAT1-mCherry (tr. ALCAT1) and grown in a glucose (5.6 mM) or galactose (10 mM) medium for 48 h. (b) Average fluorescence lifetimes *τ*_amp_ of CoxVIIIa-sEcGFP under different respiratory conditions +/- ALCAT1-mCherry overexpression. (c) Average fluorescence lifetimes *τ*_amp_ of CoxIV-sEcGFP under different respiratory conditions±ALCAT1-mCherry overexpression. *N* = 2 replicates. Data was analyzed by One-Way ANOVA with post hoc Scheffe test. Scale bars: 20 *μ*M (a).

The interaction of CL with complex I (CI), complex III (CIII), and complex IV (CIV) of the ETC is suggested to support their assembly into respiratory supercomplexes (SCs). Oxidation of CL, increased unmature CL, and a decreased overall CL level resulted in reduced SC formation, a phenomenon described in association with the Barth syndrome [32]. We wondered what effects ALCAT1 overexpression has on SC formation. To test this, we used a life cell compatible sensor for SC formation, sEcGFP fused to subunit CoxVIIIa of CIV. When SCs are formed, CoxVIIIa-sEcGFP is buried in a dense molecular environment at the interface of CI, CIII, and CIV which decreases the fluorescence lifetime *τ* of the sensor sEcGFP [28]. As a control, another subunit of CIV, CoxIV, was fused to sEcGFP. Even in a SC, the sensor sEcGFP at CoxIV is not exposed to a different environment and the fluorescence lifetime does not change. *τ* was determined by time correlated single photon counting. As depicted in Figure 6, *τ* decreased under conditions of higher respiration (Galactose conditions), indicating more SCs. In ALCAT1 overexpressing cells, this effect was not observed; the mean *τ* was the same in glucose and galactose and similar to the low glucose conditions of HeLa cells. Apparently, ALCAT1 levels above a certain threshold hampered SC formation, probably mediated by CL.

## 4. Discussion

Here, we describe the effects of elevated ALCAT1 levels on mitochondrial bioenergetics, ROS formation, and respiratory supercomplex formation. Elevated ALCAT1 levels were obtained by the expression of an exogenous plasmid. We could show that ALCAT1, when artificially overexpressed, is localized at the ER but also at mitochondrial membranes as described before [8, 27]. The colocalization of the ALCAT1-mCherry signal with the ER marker KDEL together with the findings of [27], who showed that ALCAT1 enzyme activity is higher in subcellular fractions of MAMs than of mitochondria, point to a preferential localization in the MAMs. However, this localization is not exclusively as mitochondria themselves still exhibit ALCAT1 activity [27] and in the liver of mice, a mass spectrometry-based proteomic analysis of the MAMs did not identify ALCAT1 at all [33]. A plausible, but still to be tested hypothesis is that critical high ALCAT1 levels such as induced by artificial overexpression result in ALCAT1 accumulation in MAMs. In contrast, localization in lysosomes is probably a general overexpression artifact.

We wondered whether ALCAT1 levels would naturally change under different metabolic conditions. Different metabolic conditions were induced by the change of sugars in the medium. A switch from glucose to galactose is known to upregulate OXPHOS [30] by changing the activity and/or expression of proteins involved in aerobic energy metabolism [34]. The determination of endogenous ALCAT1 protein levels by immunoblotting showed a high variability, though. For this reason, our data on endogenous ALCAT1 levels in cells do not allow a conclusive statement as to whether ALCAT1 levels change under different metabolic conditions. When glucose was replaced by galactose, mitochondrial respiration was stimulated and ATP was preferably produced by aerobic phosphorylation. Accordingly, we found that in galactose ROS production was enhanced, in particular in ALCAT1 overexpressing cells. In this context, it is an important observation that artificially enhanced ALCAT1 negatively affected respiratory supercomplex (SC) assembly. CL binds to the ETC complexes I, III, and IV and the ADP/ATP carriers. To maintain full enzymatic function, CIV requires two associated CL molecules, while CIII also needs CL to maintain its quaternary structure and function [35]. It was reported that membrane-embedded transmembrane helices form a cavity in the transmembrane region of CIII [36]. The cavity, which is located at the CIII/CIV interface, is filled with CL and phosphatidylethanolamine. Since CL's bound to ETC complexes promote SC assembly [15, 37], CLs can be seen as a glue for supercomplexes [38]. For these reasons, it does not seem unlikely that reduced supercomplex formation in ALCAT1 overexpressing cells is the result of altered cardiolipin levels or specifically modified CLs that do not provide the glue for supercompex formation anymore. Further studies should directly address how and to to which extend cardiolipin species or cardiolipin levels are affected under enhanced ALCAT1 conditions. These studies specifically should focus on the peroxidation of CL.

## Data Availability

Data will be made available upon request.

## Supplementary Materials

Supplementary Materials Suppl. Figure 1: Seahorse XF Cell Mito Stress Test Profile. The serial injection of ETC complex inhibitors enables the measurement of basal respiration, proton leak, maximal respiration, and nonmitochondrial respiration (XF Cell Mito Stress Test Kit User Guide). Suppl. Figure S3: Western Blot analysis of tr. ALCAT1-2A-GFP-NLS. (A) Western Blot result of VDAC and ALCAT1 in HeLa wt and tr. ALCAT-2A-GFP-NLS cells in glucose 48 h after transfection. (B) Quantitative analysis of ALCAT1 protein expression after normalization with VDAC. The bands in the middle are not included as they belong to another experiment. Table 1: primary antibodies used for Western Blot analysis.
