# Supplementary material for: ALCAT1 Overexpression Affects Supercomplex Formation and Increases ROS in Respiring Mitochondria
Source: Oxid Med Cell Longev. 2019 Dec 6;2019:9186469. doi: 10.1155/2019/9186469 (PMC6925921; doi:10.1155/2019/9186469)
Supplement: Supplementary Materials — Suppl. Figure 1: Seahorse XF Cell Mito Stress Test Profile. The serial injection of ETC complex inhibitors enables the measurement of basal respiration, proton leak, maximal respiration, and nonmitochondrial respiration (XF Cell Mito Stress Test Kit User Guide). Suppl. Figure S3: Western Blot analysis of tr. ALCAT1-2A-GFP-NLS. (A) Western Blot result of VDAC and ALCAT1 in HeLa wt and tr. ALCAT-2A-GFP-NLS cells in glucose 48 h after transfection. (B) Quantitative analysis of ALCAT1 protein expression after normalization with VDAC. The bands in the middle are not included as they belong to another experiment. Table 1: primary antibodies used for Western Blot analysis. [file 9186469.f1.pdf]

Artificial ALCAT1 overexpression affects supercomplex formation and increases ROS in respiring mitochondria

Bettina Rieger*[1], Adéla Krajčová [1,2], Patrick Duwe[1], Karin B. Busch*[1]

Supplementary information

Co-localization analysis of ALCAT1. The quantification of the co-localization was performed using the Pearson coefficient, with a value of +1 for perfect correlation and -1 for perfect anticorrelation. According to these values, the proportion of ALCAT1 co-localization with lysosomes was significantly higher than the co-localization of ALCAT1 and mitochondria or lysosomes and mitochondria (Fig. S1B-C). Thresholding only made a significant difference for the last two groups (ALCAT1 and mitochondria compared to lysosomes and mitochondria), which were equal. In addition to the Pearson coefficient, the estimated split Manders colocalization coefficients with a value of +1 for perfect correlation and 0 for perfect anticorrelation were also analyzed (Fig. S1D-E). There is a coefficient for each of the two channels indicating the proportion of the signal in one channel colocalized with the other channel. In this case, there is not much difference between the two channels. While the Pearson coefficient uses the deviation from the mean, the Manders coefficients use the absolute intensities. With these coefficients, the overlap between ALCAT1 and lysosomes is still quite high. However, the overlap between ALCAT1 and mitochondria is higher in this case, while according to this analysis the overlap between lysosomes and mitochondria is lower (Fig. S1D-E).

Seahorse XF Cell Mito Stress Test Kit. The Seahorse XF Cell Mito Stress Test Kit enables metabolic profiling of mitochondria measuring the oxygen consumption rate (OCR) and extracellular acidification rate (ECAR). It uses sequential injection of inhibitors (oligomycin, FCCP, rotenone/antimycin A) of the ETC complexes to measure basal respiration, proton leak, ATP production and non-mitochondrial respiration (Fig. S2). After measuring the basal respiration, oligomycin, an inhibitor of the ATP synthase, is injected and causes a decreased OCR that characterizes the ATP-linked mitochondrial respiration. FCCP is an uncoupling compound that intercepts the membrane potential so that the electron flow through the ETC complexes is faster and the maximal amount of oxygen is reduced by CIV. The measured OCR shows the maximal respiration. The injection of rotenone and antimycin A inhibits the activity of CI and CIII. As a result, no electrons are transported by the ETC and the mitochondrial respiration shuts down. This enables the measurement of non-mitochondrial respiration. Moreover, the ratio of ATP production to basal respiration represents the coupling efficiency of the mitochondrial respiration (XF Cell Mito Stress Test Kit User Guide).

Determination of mitochondrial membrane potential. Tetramethylrhodamine ethyl ester (TMRE) is a fluorescent and lipophilic cation that accumulates within mitochondria dependent on the negative mitochondrial membrane potential (MMP). Consequently, a higher mitochondrial fluorescence indicates a more negative MMP (O'Reilly et al., 2003). The cells were incubated in medium containing 7 nM TMRE under normal growth conditions (37 °C, 5 % CO_2). After 30 min of incubation with the dye, the samples were used for fluorescence microscopic analysis (ex. 559 nm, em. 650-710 nm). The cells were imaged using the following settings: $\lambda_{ex.}$ 579 nm, $\lambda_{em.}$ 590-610 nm.

Supplementary Figures

Supplementary Figure S1

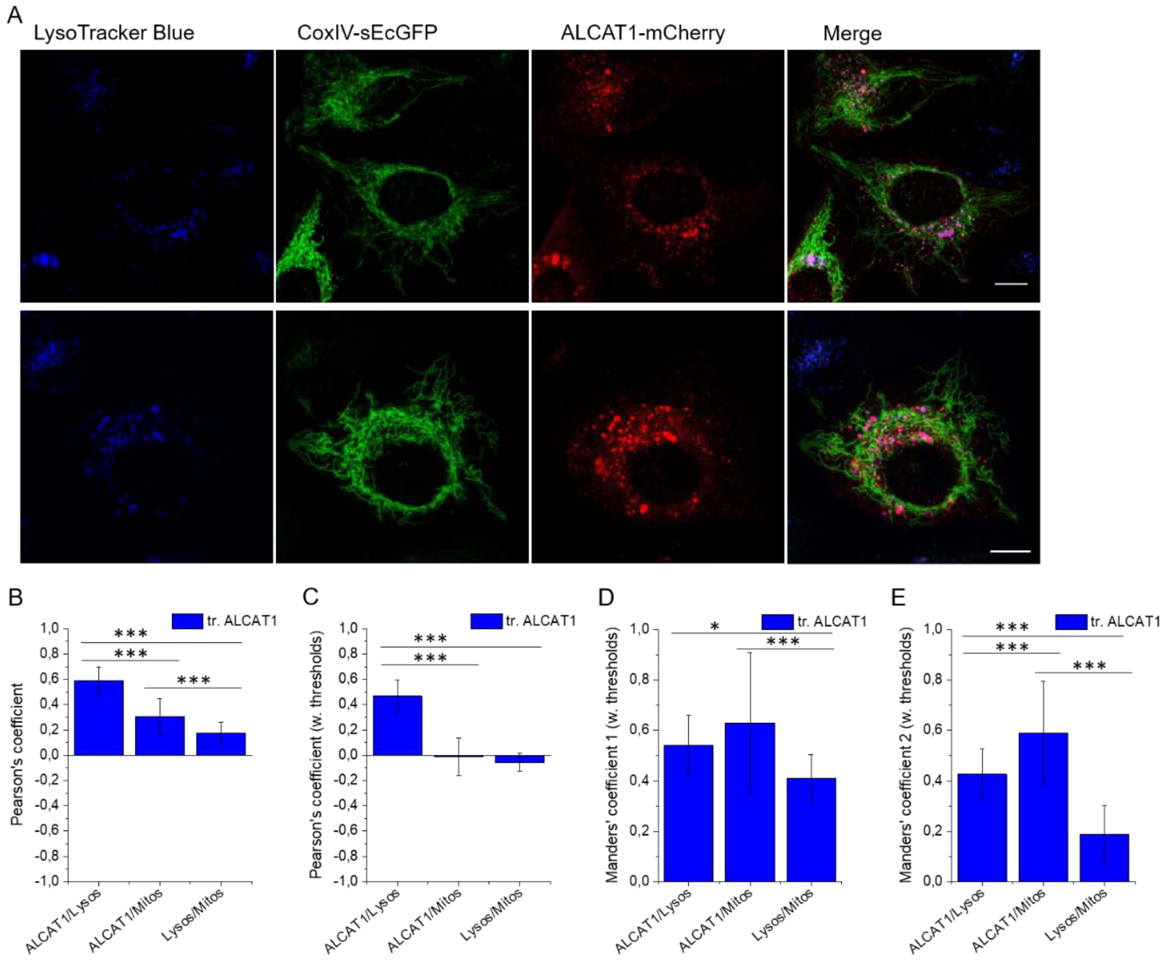

Suppl. Figure 1. Intracellular localization of overexpressed ALCAT1 in HeLa cells. (A) Triple color fluorescence microscope analysis of transiently transfected HeLa cells expressing the mitochondrial protein CoxIV-sEcGFP and ALCAT1-mCherry, stained with LysoTracker blue™. The images were processed with ImageJ to enhance the contrast. (B) Pearson's coefficients and (C) Pearson's coefficients using thresholds for both channels and (D-E) thresholded Manders' split colocalization coefficients for Co-localization analysis (N=2, 21 cells). The ImageJ plugin "Colocalization threshold" was used for calculation of the coefficients, automatically determining thresholds for each channel. One Way ANOVA with post hoc Tukey (***P < 0,001; **P < 0.01;*P < 0.05), SD are shown. Scale bars: 10 µM (A).

Supplementary Figure S2

Seahorse XF Cell Mito Stress Test Profile

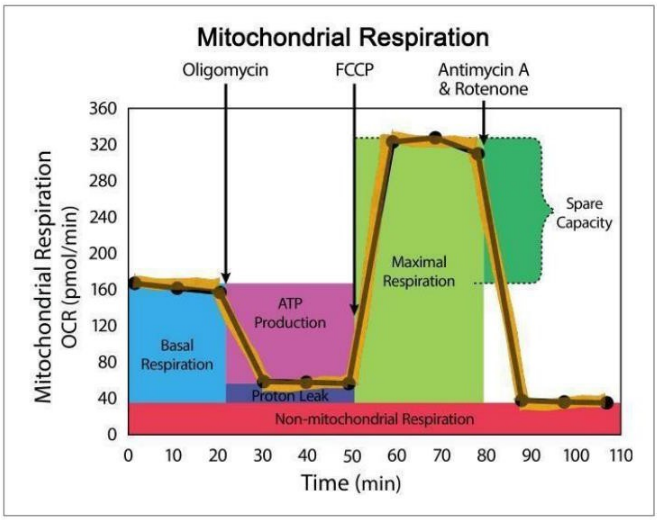

Suppl. Figure 2. Seahorse XF Cell Mito Stress Test Profile. The serial injection of ETC complex inhibitors enables the measurement of basal respiration, proton leak, maximal respiration and non-mitochondrial respiration. (XF Cell Mito Stress Test Kit User Guide)

Supplementary Figure S3

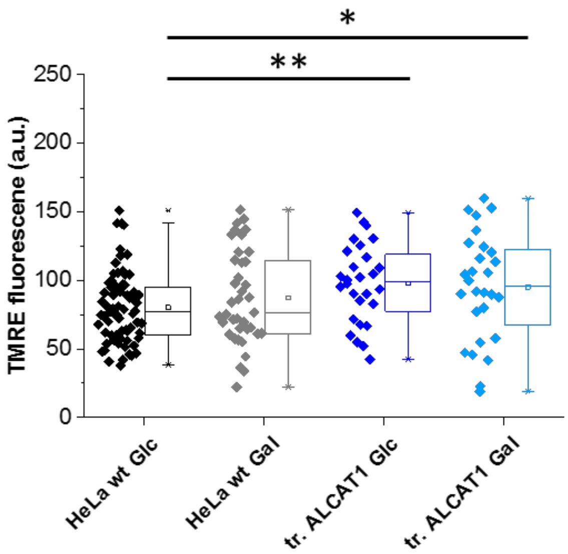

Suppl. Figure S3. Mitochondrial membrane potential in ALCAT overexpressing cells. Mitochondria in cells were stained with TMRE: (λex. 559 nm, λem. 650-710 nm. (A and B) Quantitative analysis of fluorescence intensities displayed as box plots with the measured fluorescence intensities of 20-30 measured cells on the left. The experiments were performed at RT without temperature control. N=2 (****P* < 0,001; ***P* < 0.01;**P* < 0.05).

Supplementary Information

Table 1. Primary antibodies used for Western Blot analysis

Name	Species reactivity	Host	poly/mono	kDa	Dilution for WB	Company
Anti-ALCAT1	Human	Rabbit	poly	49	1:2000	abcam
Anti-ATP5b [Suβ]	Human, Rat, Mouse	Rabbit	poly	56	1:2000	proteintech
Anti-ATP5I [Sue]	Human, Rat, Mouse	Rabbit	poly	8	1:500	abcam
Anti-VDAC	Human, Rat, Mouse	Rabbit	poly	32	1:2000	Cell signaling

The peroxidase-conjugated AffiniPure Goat Anti-Rabbit IgG (H+L) (Jackson ImmunoResearch, dianova) was used as a secondary antibody at a final dilution of 1:2000.

Gene sequence of pSems-ALCAT1-2A-GFP-NL

Molecule Features

Molecule: pSems-ALCAT1-2A-GFP-NLS, 7301 bps DNA Circular

File Name: pSems-ALCAT1-2A-GFP-NLS.cm5, dated 23 Feb 2016

Description:

Start	End	Name	Feature Key	Draw
921	2162	ALCAT1		Gene
2166	2231	2A		Gene
2232	2945	EGFP		Gene
2952	3020	NLS		Gene
3988	4788	Neomycin		Gene

GACGGATCGGGAGATCTCCCGATCCCCTATGGTCGACTCT

CAGTACAATC

TGCTCTGATGCCGCATAGTTAAGCCAGTATCTGCTCCCTGC

TTGTGTGTT

GGAGGTCGCTGAGTAGTGCGCGAGCAAAATTTAAGCTAC

AACAAGGCAAG

GCTTGACCGACAATTGCATGAAGAATCTGCTTAGGGTTAG

GCGTTTTGCG

CTGCTTCGCGATGTACGGGCCAGATATACGCGTTGACATT

GATTATTGAC

TAGTTATTAATAGTAATCAATTACGGGGTCATTAGTTCATA

GCCCATATA

TGGAGTTCCGCGTTACATAACTTACGGTAAATGGCCCGCC

TGGCTGACCG

CCCAACGACCCCCGCCCATTGACGTCAATAATGACGTATGT

TCCCATAGT

AACGCCAATAGGGACTTTCCATTGACGTCAATGGGTGGAC

TATTTACGGT

AAACTGCCCACTTGGCAGTACATCAAGTGTATCATATGCCA

AGTACGCCC

CCTATTGACGTCAATGACGGTAAATGGCCCGCCTGGCATT

ATGCCCAGTA

CATGACCTTATGGGACTTTCCTACTTGGCAGTACATCTACG

TATTAGTCA

TCGCTATTACCATGGTGATGCGGTTTTGGCAGTACATCAAT

GGGCGTGGA

TAGCGGTTTGACTCACGGGGATTTCCAAGTCTCCACCCCAT

TGACGTCAA

TGGGAGTTTGTTTTGGCACCAAAATCAACGGGACTTTCCA

AAATGTCGTA

ACAACTCCGCCCCATTGACGCAAATGGGCGGTAGGCGTGT

ACGGTGGGAG

GTCTATATAAGCAGAGCTCTCTGGCTAACTAGAGAACCCA

CTGCTTACTG

GCTTATCGAAATTAATACGACTCACTATAGGGAGACCCAA

GCTTGGTACC

GAGCTCGGACCGGTGCTAGCATGCATTCCAGGGGAAGGGAAATTGTGGTG
CTTCTGAACCCATGGTCAATTAACGAGGCAGTTTCTAGCTACTGCACGTA
CTTCATAAAGCAGGACTCTAAAAGCTTTGGAATCATGGTGTCATGGAAAG
GGATTTACTTTATACTGACTCTGTTTTGGGGAAGCTTTTTTGGAAGCATT
TTCATGCTGAGTCCCTTTTTACCTTTGATGTTTGTAAACCCATCTTGGTA
TCGCTGGATCAACAACCGCCTTGTGGCAACATGGCTCACCCTACCTGTGG
CATTATTGGAGACCATGTTTGGTGTAAAAGTGATTATAACTGGGGATGCA
TTTGTTCCTGGAGAAAGAAGTGTCATTATCATGAACCATCGGACAAGAAT
GGACTGGATGTTCCTGTGGAATTGCCTGATGCGATATAGCTACCTCAGAT
TGGAGAAAATTTGCCTCAAAGCGAGTCTCAAAGGTGTTCCTGGATTTGGT
TGGGCCATGCAGGCTGCTGCCTATATCTTCATTCATAGGAAATGGAAGGA

TGACAAGAGCCATTTCGAAGACATGATTGATTACTTTTGTGATATTCACG
AACCACTTCAACTCCTCATATTCCCAGAAGGGACTGATCTCACAGAAAAC
AGCAAGTCTCGAAGTAATGCATTTGCTGAAAAAAATGGACTTCAGAAATA
TGAATATGTTTTACATCCAAGAACTACAGGCTTTACTTTTGTGGTAGACC
GTCTAAGAGAAGGTAAGAACCTTGATGCTGTCCATGATATCACTGTGGCG
TATCCTCACAACATTCCTCAATCAGAGAAGCACCTCCTCCAAGGAGACTT
TCCCAGGGAAATCCACTTTCACGTCCACCGGTATCCAATAGACACCCTCC
CCACATCCAAGGAGGACCTTCAACTCTGGTGCCACAAACGGTGGGAAGAG
AAAGAAGAGAGGCTGCGTTCCTTCTATCAAGGGGAGAAGAATTTTTATTT
TACCGGACAGAGTGTCATTCCACCTTGCAAGTCTGAACTCAGGGTCCTTG
TGGTCAAATTGCTCTCTATACTGTATTGGACCCTGTTCAGCCCTGCAATG
TGCCTACTCATATATTTGTACAGTCTTGTTAAGTGGTATTTTATAATCAC
CATTGTAATCTTTGTGCTGCAAGAGAGAATATTTGGTGGACTGGAGATCA
TAGAACTTGCATGTTACCGACTTTTACACAAACAGCCACATTTAAATTCA
AAGAAAAATGAGCTCGAGGGCAGTGGAGAGGGCAGAGG

AAGTCTGCTAAC

ATGCGGTGACGTCGAGGAGAATCCTGGCCCAGTGAGCAA

GGGCGAGGAGC

TGTTCACCGGGGTGGTGCCCATCCTGGTCGAGCTGGACGGCGACGTAAAC
GGCCACAAGTTCAGCGTGTCCGGCGAGGGCGAGGGCGATGCCACCTACGG
CAAGCTGACCCTGAAGTTCATCTGCACCACCGGCAAGCTGCCCGTGCCCT
GGCCCACCCTCGTGACCACCCTGACCTACGGCGTGCAGTGCTTCAGCCGC
TACCCCGACCACATGAAGCAGCACGACTTCTTCAAGTCCGCCATGCCCGA
AGGCTACGTCCAGGAGCGCACCATCTTCTTCAAGGACGACGGCAACTACA
AGACCCGCGCCGAGGTGAAGTTCGAGGGCGACACCCTGGTGAACCGCATC
GAGCTGAAGGGCATCGACTTCAAGGAGGACGGCAACATCCTGGGGCACAA
GCTGGAGTACAACTACAACAGCCACAACGTCTATATCATGGCCGACAAGC
AGAAGAACGGCATCAAGGTGAACTTCAAGATCCGCCACAACATCGAGGAC
GGCAGCGTGCAGCTCGCCGACCACTACCAGCAGAACACCCCCATCGGCGA
CGGCCCCGTGCTGCTGCCCGACAACCACTACCTGAGCACCCAGTCCGCCC
TGAGCAAAGACCCCAACGAGAAGCGCGATCACATGGTCCTGCTGGAGTTC
GTGACCGCCGCCGGGATCACTCTCGGCATGGACGAGCTGTACAAGGCGGC
CCCAAAGAAAAAGCGTAAAGTTGATCCAAAAAAGAAGAGAAAGGTAGATC
CAAAGAAAAAGCGTAAAGTTTAAGCGGCCGCATAGATAA

CTGATCCAGTG

TGCTGGAATTAATTCGCTGTCTGCGAGGGCCAGCTGTTGG

GGTGAGTACT

CCCTCTCAAAAGCGGGCATGACTTCTGCGCTAAGATTGTC

AGTTTCCAAA

AACGAGGAGGATTTGATATTCACCTGGCCCGCGGTGATGC

CTTTGAGGGT

GGCCGCGTCCATCTGGTCAGAAAAGACAATCTTTTTGTTGT
CAAGCTTGA

GGTGTGGCAGGCTTGAGATCTGGCCATACACTTGAGTGAC
AATGACATCC

ACTTTGCCTTTCTCTCCACAGGTGTCCACTCCCAGGTCCAA
CTGCAGGTC

GAGCATGCATCTAGGGCGGCCAATTCCGCCCCTCTCCCTCC
CCCCCCCCT

AACGTTACTGGCCGAAGCCGCTTGGAATAAGGCCGGTGT
GCGTTTGTCTA

TATGTTATTTTCCACCATATTGCCGTCTTTTGGCAATGTGAG
GGCCCGGA

AACCTGGCCCTGTCTTCTTGACGAGCATTCCTAGGGGTCTT
TCCCCTCTC

GCCAAAGGAATGCAAGGTCTGTTGAATGTCGTGAAGGAA
GCAGTTCCTCT

GGAAGCTTCTTGAAGACAAACAACGTCTGTAGCGACCCTT
TGCAGGCAGC

GGAACCCCCCACCTGGCGACAGGTGCCTCTGCGGCCAAAA
GCCACGTGTA

TAAGATACACCTGCAAAGGCGGCACAACCCCAGTGCCACG
TTGTGAGTTG

GATAGTTGTGGAAAGAGTCAAATGGCTCTCCTCAAGCGTA
TTCAACAAGG

GGCTGAAGGATGCCCAGAAGGTACCCCATTGTATGGGATC
TGATCTGGGG

CCTCGGTGCACATGCTTTACATGTGTTTAGTCGAGGTTAAA
AAAACGTCT

AGGCCCCCCGAACCACGGGGACGTGGTTTTCCTTTGAAAA
ACACGATGAT

AAGCTTGCCACAACCCGGGATAATTCCTGCAGCCAATATG

GGATCGGCCA

TTGAACAAGATGGATTGCACGCAGGTTCTCCGGCCGCTTG

GGTGGAGAGG

CTATTCGGCTATGACTGGGCACAACAGACAATCGGCTGCT

CTGATGCCGC

CGTGTTCCGGCTGTCAGCGCAGGGGCGCCCGGTTCTTTTT

GTCAAGACCG

ACCTGTCCGGTGCCCTGAATGAACTGCAGGACGAGGCAG

CGCGGCTATCG

TGGCTGGCCACGACGGGCGTTCCTTGCGCAGCTGTGCTCG

ACGTTGTCAC

TGAAGCGGGAAGGGACTGGCTGCTATTGGGCGAAGTGCC

GGGGCAGGATC

TCCTGTCATCTCACCTTGCTCCTGCCGAGAAAGTATCCATC

ATGGCTGAT

GCAATGCGGCGGCTGCATACGCTTGATCCGGCTACCTGCC

CATTCGACCA

CCAAGCGAAACATCGCATCGAGCGAGCACGTACTCGGATG

GAAGCCGGTC

TTGTCGATCAGGATGATCTGGACGAAGAGCATCAGGGGC

TCGCGCCAGCC

GAACTGTTCGCCAGGCTCAAGGCGCGCATGCCCGACGGC

GATGATCTCGT

CGTGACCCATGGCGATGCCTGCTTGCCGAATATCATGGTG

GAAAATGGCC

GCTTTTCTGGATTCATCGACTGTGGCCGGCTGGGTGTGGC

GGACCGCTAT

CAGGACATAGCGTTGGCTACCCGTGATATTGCTGAAGAGC

TTGGCGGCGA

ATGGGCTGACCGCTTCCTCGTGCTTTACGGTATCGCCGCTC
CCGATTCGC

AGCGCATCGCCTTCTATCGCCTTCTTGACGAGTTCTTCTGA
GGGGATCAA

TTCTCTAGATAACTGATCATAATCAGCCATACCACATTTGT
AGAGGTTTT

ACTTGCTTTAAAAAACCTCCCACACCTCCCCCTGAACCTGA
AACATAAAA

TGAATGCAATTGTTGTTGTTAACTTGTTTATTGCAGCTTATA
ATGGTTAC

AAATAAAGCAATAGCATCACAAATTTCACAAATAAAGCAT
TTTTTTCACT

GCATTCTAGTTGTGGTTTGTCCAAACTCATCAATGTATCTT
AACGCGTCG

AGTGCATTCTAGTTGTGGTTTGTCCAAACTCATCAATGTAT
CTTATCATG

TCTGTATACCGTCGACCTCTAGCTAGAGCTTGGCGTAATCA
TGGTCATAG

CTGTTTCCTGTGTGAAATTGTTATCCGCTCACAATTCCACAC
AACATACG

AGCCGGAAGCATAAAGTGTAAAGCCTGGGGTGCCTAATG
AGTGAGCTAAC

TCACATTAATTGCGTTGCGCTCACTGCCCGCTTTCCAGTCG
GGAAACCTG

TCGTGCCAGCTGCATTAATGAATCGGCCAACGCGCGGGGA
GAGGCGGTTT

GCGTATTGGGCGCTCTTCCGCTTCCTCGCTCACTGACTCGC
TGCGCTCGG

TCGTTCGGCTGCGGCGAGCGGTATCAGCTCACTCAAAGGC
GGTAATACGG

TTATCCACAGAATCAGGGGATAACGCAGGAAAGAACATGT
GAGCAAAAGG

CCAGCAAAAGGCCAGGAACCGTAAAAAGGCCGCGTTGCT
GGCGTTTTTCC

ATAGGCTCCGCCCCCCTGACGAGCATCACAAAAATCGACG
CTCAAGTCAG

AGGTGGCGAAACCCGACAGGACTATAAAGATACCAGGCG
TTTCCCCCTGG

AAGCTCCCTCGTGCGCTCTCCTGTTCCGACCCTGCCGCTTA
CCGGATACC

TGTCCGCCTTTCTCCCTTCGGGAAGCGTGGCGCTTTCTCAA
TGCTCACGC

TGTAGGTATCTCAGTTCGGTGTAGGTCGTTCGCTCCAAGCT
GGGCTGTGT

GCACGAACCCCCCGTTCAGCCCGACCGCTGCGCCTTATCC
GGTAACTATC

GTCTTGAGTCCAACCCGGTAAGACACGACTTATCGCCACT
GGCAGCAGCC

ACTGGTAACAGGATTAGCAGAGCGAGGTATGTAGGCGGT
GCTACAGAGTT

CTTGAAGTGGTGGCCTAACTACGGCTACACTAGAAGGACA
GTATTTGGTA

TCTGCGCTCTGCTGAAGCCAGTTACCTTCGGAAAAAGAGT
TGGTAGCTCT

TGATCCGGCAAACAAACCACCGCTGGTAGCGGTGGTTTTT
TTGTTTGCAA

GCAGCAGATTACGCGCAGAAAAAAAGGATCTCAAGAAGA
TCCTTTGATCT

TTTCTACGGGGTCTGACGCTCAGTGGAACGAAAACTCACG
TTAAGGGATT

TTGGTCATGAGATTATCAAAAAGGATCTTCACCTAGATCCT
TTTAAATTA

AAAATGAAGTTTTAAATCAATCTAAAGTATATATGAGTAAA
CTTGGTCTG

ACAGTTACCAATGCTTAATCAGTGAGGCACCTATCTCAGC
GATCTGTCTA

TTTCGTTCATCCATAGTTGCCTGACTCCCCGTCGTGTAGAT
AACTACGAT

ACGGGAGGGCTTACCATCTGGCCCCAGTGCTGCAATGATA
CCGCGAGACC

CACGCTCACCGGCTCCAGATTTATCAGCAATAAACCAGCCA
GCCGGAAGG

GCCGAGCGCAGAAGTGGTCCTGCAACTTTATCCGCCTCCA
TCCAGTCTAT

TAATTGTTGCCGGGAAGCTAGAGTAAGTAGTTCGCCAGTT
AATAGTTTGC

GCAACGTTGTTGCCATTGCTACAGGCATCGTGGTGTCACG
CTCGTCGTTT

GGTATGGCTTCATTCAGCTCCGGTTCCCAACGATCAAGGC
GAGTTACATG

ATCCCCCATGTTGTGCAAAAAAGCGGTTAGCTCCTTCGGTC
CTCCGATCG

TTGTCAGAAGTAAGTTGGCCGCAGTGTTATCACTCATGGTT
ATGGCAGCA

CTGCATAATTCTCTTACTGTCATGCCATCCGTAAGATGCTTT
TCTGTGAC

TGGTGAGTACTCAACCAAGTCATTCTGAGAATAGTGTATG
CGGCGACCGA

GTTGCTCTTGCCCGGCGTCAATACGGGATAATACCGCGCC
ACATAGCAGA

ACTTTAAAAGTGCTCATCATTGGAAAACGTTCTTCGGGGC

GAAAACTCTC

AAGGATCTTACCGCTGTTGAGATCCAGTTCGATGTAACCC

ACTCGTGCAC

CCAACTGATCTTCAGCATCTTTTACTTTCACCAGCGTTTCTG

GGTGAGCA

AAAACAGGAAGGCAAAATGCCGCAAAAAAGGGAATAAG

GGCGACACGGAA

ATGTTGAATACTCATACTCTTCCTTTTTCAATATTATTGAAG

CATTTATC

AGGGTTATTGTCTCATGAGCGGATACATATTTGAATGTATT

TAGAAAAAT

AAACAAATAGGGGTTCCGCGCACATTTCCCCGAAAAGTGC

CACCTGACGT C

Plasmid map and gene sequence of pSems-ALCAT1-HA

Molecule Features

Molecule: pSems-ALCAT1-HAtag, 6476 bps DNA Circular

File Name: pSems-ALCAT1-HAtag.cm5, dated 11 Feb 2016 Description: Ligation of product ALCAT1* into pSems-1-26-Halo7-AN-HAtag*

Start	End	Name	Feature Key	Draw
921	2162	ALCAT1		Gene
2169	2195	HA-tag		Gene
3163	3963	Neomycin		Gene

GACGGATCGGGAGATCTCCCGATCCCCTATGGTCGACTCT

CAGTACAATC

TGCTCTGATGCCGCATAGTTAAGCCAGTATCTGCTCCCTGC

TTGTGTGTT

GGAGGTCGCTGAGTAGTGCGCGAGCAAAATTTAAGCTAC

AACAAGGCAAG

GCTTGACCGACAATTGCATGAAGAATCTGCTTAGGGTTAG

GCGTTTTGCG

CTGCTTCGCGATGTACGGGCCAGATATACGCGTTGACATT

GATTATTGAC

TAGTTATTAATAGTAATCAATTACGGGGTCATTAGTTCATA

GCCCATATA

TGGAGTTCCGCGTTACATAACTTACGGTAAATGGCCCGCC

TGGCTGACCG

CCCAACGACCCCCGCCCATTGACGTCAATAATGACGTATGT

TCCCATAGT

AACGCCAATAGGGACTTTCCATTGACGTCAATGGGTGGAC

TATTTACGGT

AAACTGCCCACTTGGCAGTACATCAAGTGTATCATATGCCA

AGTACGCCC

CCTATTGACGTCAATGACGGTAAATGGCCCGCCTGGCATT

ATGCCCAGTA

CATGACCTTATGGGACTTTCCTACTTGGCAGTACATCTACG

TATTAGTCA

TCGCTATTACCATGGTGATGCGGTTTTGGCAGTACATCAAT

GGGCGTGGA

TAGCGGTTTGACTCACGGGGATTTCCAAGTCTCCACCCCAT

TGACGTCAA

TGGGAGTTTGTTTTGGCACCAAAATCAACGGGACTTTCCA

AAATGTCGTA

ACAACTCCGCCCCATTGACGCAAATGGGCGGTAGGCGTGT

ACGGTGGGAG

GTCTATATAAGCAGAGCTCTCTGGCTAACTAGAGAACCCA

CTGCTTACTG

GCTTATCGAAATTAATACGACTCACTATAGGGAGACCCAA

GCTTGGTACC

GAGCTCGGACCGGTGCTAGCATGCATTCCAGGGGAAGGGAAATTGTGGTG
CTTCTGAACCCATGGTCAATTAACGAGGCAGTTTCTAGCTACTGCACGTA
CTTCATAAAGCAGGACTCTAAAAGCTTTGGAATCATGGTGTCATGGAAAG
GGATTTACTTTATACTGACTCTGTTTTGGGGAAGCTTTTTTGGAAGCATT
TTCATGCTGAGTCCCTTTTTACCTTTGATGTTTGTAAACCCATCTTGGTA
TCGCTGGATCAACAACCGCCTTGTGGCAACATGGCTCACCCTACCTGTGG
CATTATTGGAGACCATGTTTGGTGTAAAAGTGATTATAACTGGGGATGCA
TTTGTTCCTGGAGAAAGAAGTGTCATTATCATGAACCATCGGACAAGAAT
GGACTGGATGTTCCTGTGGAATTGCCTGATGCGATATAGCTACCTCAGAT
TGGAGAAAATTTGCCTCAAAGCGAGTCTCAAAGGTGTTCCTGGATTTGGT
TGGGCCATGCAGGCTGCTGCCTATATCTTCATTCATAGGAAATGGAAGGA
TGACAAGAGCCATTTCGAAGACATGATTGATTACTTTTGTGATATTCACG
AACCACTTCAACTCCTCATATTCCCAGAAGGGACTGATCTCACAGAAAAC
AGCAAGTCTCGAAGTAATGCATTTGCTGAAAAAAATGGACTTCAGAAATA
TGAATATGTTTTACATCCAAGAACTACAGGCTTTACTTTTGTGGTAGACC
GTCTAAGAGAAGGTAAGAACCTTGATGCTGTCCATGATATCACTGTGGCG
TATCCTCACAACATTCCTCAATCAGAGAAGCACCTCCTCCAAGGAGACTT
TCCCAGGGAAATCCACTTTCACGTCCACCGGTATCCAATAGACACCCTCC
CCACATCCAAGGAGGACCTTCAACTCTGGTGCCACAAACGGTGGGAAGAG
AAAGAAGAGAGGCTGCGTTCCTTCTATCAAGGGGAGAAGAATTTTTATTT

TACCGGACAGAGTGTCATTCCACCTTGCAAGTCTGAACTCAGGGTCCTTG
TGGTCAAATTGCTCTCTATACTGTATTGGACCCTGTTCAGCCCTGCAATG
TGCCTACTCATATATTTGTACAGTCTTGTTAAGTGGTATTTTATAATCAC
CATTGTAATCTTTGTGCTGCAAGAGAGAATATTTGGTGGACTGGAGATCA
TAGAACTTGCATGTTACCGACTTTTACACAAACAGCCACATTTAAATTCA
AAGAAAAATGAGCTCGAGTATCCATATGACGTCCCAGACTATGCCTAGGC
GGCCGCATAGATAACTGATCCAGTGTGCTGGAATTAATTC

GCTGTCTGCG

AGGGCCAGCTGTTGGGGTGAGTACTCCCTCTCAAAAGCGG

GCATGACTTC

TGCGCTAAGATTGTCAGTTTCCAAAAACGAGGAGGATTTG

ATATTCACCT

GGCCCGCGGTGATGCCTTTGAGGGTGGCCGCGTCCATCTG

GTCAGAAAAG

ACAATCTTTTTGTTGTCAAGCTTGAGGTGTGGCAGGCTTGA

GATCTGGCC

ATACACTTGAGTGACAATGACATCCACTTTGCCTTTCTCTCC

ACAGGTGT

CCACTCCCAGGTCCAACTGCAGGTCGAGCATGCATCTAGG

GCGGCCAATT

CCGCCCCTCTCCCTCCCCCCCCCCTAACGTTACTGGCCGAA

GCCGCTTGG

AATAAGGCCGGTGTGCGTTTGTCTATATGTTATTTTCCACC

ATATTGCCG

TCTTTTGGCAATGTGAGGGCCCGGAAACCTGGCCCTGTCT

TCTTGACGAG

CATTCCTAGGGGTCTTTCCCCTCTCGCCAAAGGAATGCAAG

GTCTGTTGA

ATGTCGTGAAGGAAGCAGTTCCTCTGGAAGCTTCTTGAAG

ACAAACAACG

TCTGTAGCGACCCTTTGCAGGCAGCGGAACCCCCCACCTG

GCGACAGGTG

CCTCTGCGGCCAAAAGCCACGTGTATAAGATACACCTGCA

AAGGCGGCAC

AACCCCAGTGCCACGTTGTGAGTTGGATAGTTGTGGAAAG

AGTCAAATGG

CTCTCCTCAAGCGTATTCAACAAGGGGCTGAAGGATGCCC

AGAAGGTACC

CCATTGTATGGGATCTGATCTGGGGCCTCGGTGCACATGC

TTTACATGTG

TTTAGTCGAGGTTAAAAAAACGTCTAGGCCCCCCGAACCA

CGGGGACGTG

GTTTTCCTTTGAAAAACACGATGATAAGCTTGCCACAACCC

GGGATAATT

CCTGCAGCCAATATGGGATCGGCCATTGAACAAGATGGAT

TGCACGCAGG

TTCTCCGGCCGCTTGGGTGGAGAGGCTATTCGGCTATGAC

TGGGCACAAC

AGACAATCGGCTGCTCTGATGCCGCCGTGTTCCGGCTGTC

AGCGCAGGGG

CGCCCGGTTCTTTTTGTCAAGACCGACCTGTCCGGTGCCCT

GAATGAACT

GCAGGACGAGGCAGCGCGGCTATCGTGGCTGGCCACGAC

GGGCGTTCCTT

GCGCAGCTGTGCTCGACGTTGTCACTGAAGCGGGAAGGG

ACTGGCTGCTA

TTGGGCGAAGTGCCGGGGCAGGATCTCCTGTCATCTCACC

TTGCTCCTGC

CGAGAAAGTATCCATCATGGCTGATGCAATGCGGCGGCTG

CATACGCTTG

ATCCGGCTACCTGCCCATTCGACCACCAAGCGAAACATCG

CATCGAGCGA

GCACGTACTCGGATGGAAGCCGGTCTTGTCGATCAGGATG
ATCTGGACGA

AGAGCATCAGGGGCTCGCGCCAGCCGAACTGTTCGCCAG
GCTCAAGGCGC

GCATGCCCGACGGCGATGATCTCGTCGTGACCCATGGCGA
TGCCTGCTTG

CCGAATATCATGGTGGAAAATGGCCGCTTTTCTGGATTCAT
CGACTGTGG

CCGGCTGGGTGTGGCGGACCGCTATCAGGACATAGCGTT
GGCTACCCGTG

ATATTGCTGAAGAGCTTGGCGGCGAATGGGCTGACCGCTT
CCTCGTGCTT

TACGGTATCGCCGCTCCCGATTCGCAGCGCATCGCCTTCTA
TCGCCTTCT

TGACGAGTTCTTCTGAGGGGATCAATTCTCTAGATAACTG
ATCATAATCA

GCCATACCACATTTGTAGAGGTTTTACTTGCTTTAAAAAAC
CTCCCACAC

CTCCCCCTGAACCTGAAACATAAAATGAATGCAATTGTTGT
TGTTAACTT

GTTTATTGCAGCTTATAATGGTTACAAATAAAGCAATAGCA
TCACAAATT

TCACAAATAAAGCATTTTTTTCACTGCATTCTAGTTGTGGTT
TGTCCAAA

CTCATCAATGTATCTTAACGCGTCGAGTGCATTCTAGTTGT
GGTTTGTCC
AAACTCATCAATGTATCTTATCATGTCTGTATACCGTCGAC
CTCTAGCTA

GAGCTTGGCGTAATCATGGTCATAGCTGTTTCCTGTGTGA
AATTGTTATC

CGCTCACAATTCCACACAACATACGAGCCGGAAGCATAAA

GTGTAAAGCC

TGGGGTGCCTAATGAGTGAGCTAACTCACATTAATTGCGT

TGCGCTCACT

GCCCGCTTTCCAGTCGGGAAACCTGTCGTGCCAGCTGCAT

TAATGAATCG

GCCAACGCGCGGGGAGAGGCGGTTTGCGTATTGGGCGCT

CTTCCGCTTCC

TCGCTCACTGACTCGCTGCGCTCGGTCGTTCGGCTGCGGC

GAGCGGTATC

AGCTCACTCAAAGGCGGTAATACGGTTATCCACAGAATCA

GGGGATAACG

CAGGAAAGAACATGTGAGCAAAAGGCCAGCAAAAGGCCA

GGAACCGTAAA

AAGGCCGCGTTGCTGGCGTTTTTCCATAGGCTCCGCCCCCC

TGACGAGCA

TCACAAAAATCGACGCTCAAGTCAGAGGTGGCGAAACCCG

ACAGGACTAT

AAAGATACCAGGCGTTTCCCCCTGGAAGCTCCCTCGTGCG

CTCTCCTGTT

CCGACCCTGCCGCTTACCGGATACCTGTCCGCCTTTCTCCC

TTCGGGAAG

CGTGGCGCTTTCTCAATGCTCACGCTGTAGGTATCTCAGTT

CGGTGTAGG

TCGTTCGCTCCAAGCTGGGCTGTGTGCACGAACCCCCCGTT

CAGCCCGAC

CGCTGCGCCTTATCCGGTAACTATCGTCTTGAGTCCAACCC

GGTAAGACA

CGACTTATCGCCACTGGCAGCAGCCACTGGTAACAGGATT

AGCAGAGCGA

GGTATGTAGGCGGTGCTACAGAGTTCTTGAAGTGGTGGCC

TAACTACGGC

TACACTAGAAGGACAGTATTTGGTATCTGCGCTCTGCTGA

AGCCAGTTAC

CTTCGGAAAAAGAGTTGGTAGCTCTTGATCCGGCAAACAA

ACCACCGCTG

GTAGCGGTGGTTTTTTTGTTTGCAAGCAGCAGATTACGCG

CAGAAAAAAA

GGATCTCAAGAAGATCCTTTGATCTTTTCTACGGGGTCTGA

CGCTCAGTG

GAACGAAAACTCACGTTAAGGGATTTTGGTCATGAGATTA

TCAAAAAGGA

TCTTCACCTAGATCCTTTTAAATTAAAAATGAAGTTTTAAAT

CAATCTAA

AGTATATATGAGTAAACTTGGTCTGACAGTTACCAATGCTT

AATCAGTGA

GGCACCTATCTCAGCGATCTGTCTATTTCGTTCATCCATAG

TTGCCTGAC

TCCCCGTCGTGTAGATAACTACGATACGGGAGGGCTTACC

ATCTGGCCCC

AGTGCTGCAATGATACCGCGAGACCCACGCTCACCGGCTC

CAGATTTATC

AGCAATAAACCAGCCAGCCGGAAGGGCCGAGCGCAGAAG

TGGTCCTGCAA

CTTTATCCGCCTCCATCCAGTCTATTAATTGTTGCCGGGAA

GCTAGAGTA

AGTAGTTCGCCAGTTAATAGTTTGCGCAACGTTGTTGCCAT

TGCTACAGG

CATCGTGGTGTCACGCTCGTCGTTTGGTATGGCTTCATTCA

GCTCCGGTT

CCCAACGATCAAGGCGAGTTACATGATCCCCCATGTTGTG

CAAAAAAGCG

GTTAGCTCCTTCGGTCCTCCGATCGTTGTCAGAAGTAAGTT

GGCCGCAGT

GTTATCACTCATGGTTATGGCAGCACTGCATAATTCTCTTA

CTGTCATGC

CATCCGTAAGATGCTTTTCTGTGACTGGTGAGTACTCAACC

AAGTCATTC

TGAGAATAGTGTATGCGGCGACCGAGTTGCTCTTGCCCGG

CGTCAATACG

GGATAATACCGCGCCACATAGCAGAACTTTAAAAGTGCTC

ATCATTGGAA

AACGTTCTTCGGGGCGAAAACTCTCAAGGATCTTACCGCT

GTTGAGATCC

AGTTCGATGTAACCCACTCGTGCACCCAACTGATCTTCAGC

ATCTTTTAC

TTTCACCAGCGTTTCTGGGTGAGCAAAAACAGGAAGGCAA

AATGCCGCAA

AAAAGGGAATAAGGGCGACACGGAAATGTTGAATACTCA

TACTCTTCCTT

TTTCAATATTATTGAAGCATTTATCAGGGTTATTGTCTCATG

AGCGGATA

CATATTTGAATGTATTTAGAAAAATAAACAAATAGGGGTT

CCGCGCACAT

TTCCCCGAAAAGTGCCACCTGACGTC

Plasmid map and gene sequence of pSems-ALCAT1-mCherry

BmrI AciI

Molecule Features

Molecule: pSems-ALCAT1-mCherry, 7154 bps DNA Circular

File Name: pSems-ALCAT1-mCherry.cm5, dated 07 Jun 2016

Description:

Start	End	Name	Feature Key	Draw
921	2162	ALCAT1		Gene
2169	2876	mCherry		Gene
3841	4641	Neomycin		Gene

GACGGATCGGGAGATCTCCCGATCCCCTATGGTCGACTCT

CAGTACAATC

TGCTCTGATGCCGCATAGTTAAGCCAGTATCTGCTCCCTGC

TTGTGTGTT

GGAGGTCGCTGAGTAGTGCGCGAGCAAAATTTAAGCTAC

AACAAGGCAAG

GCTTGACCGACAATTGCATGAAGAATCTGCTTAGGGTTAG

GCGTTTTGCG

CTGCTTCGCGATGTACGGGCCAGATATACGCGTTGACATT

GATTATTGAC

TAGTTATTAATAGTAATCAATTACGGGGTCATTAGTTCATA

GCCCATATA

TGGAGTTCCGCGTTACATAACTTACGGTAAATGGCCCGCC

TGGCTGACCG

CCCAACGACCCCCGCCCATTGACGTCAATAATGACGTATGT

TCCCATAGT

AACGCCAATAGGGACTTTCCATTGACGTCAATGGGTGGAC

TATTTACGGT

AAACTGCCCACTTGGCAGTACATCAAGTGTATCATATGCCA

AGTACGCCC

CCTATTGACGTCAATGACGGTAAATGGCCCGCCTGGCATT

ATGCCCAGTA

CATGACCTTATGGGACTTTCCTACTTGGCAGTACATCTACG

TATTAGTCA

TCGCTATTACCATGGTGATGCGGTTTTGGCAGTACATCAAT

GGGCGTGGA

TAGCGGTTTGACTCACGGGGATTTCCAAGTCTCCACCCCAT

TGACGTCAA

TGGGAGTTTGTTTTGGCACCAAAATCAACGGGACTTTCCA

AAATGTCGTA

ACAACTCCGCCCCATTGACGCAAATGGGCGGTAGGCGTGT

ACGGTGGGAG

GTCTATATAAGCAGAGCTCTCTGGCTAACTAGAGAACCCA

CTGCTTACTG

GCTTATCGAAATTAATACGACTCACTATAGGGAGACCCAA

GCTTGGTACC

GAGCTCGGACCGGTGCTAGCATGCATTCCAGGGGAAGGGAAATTGTGGTG
CTTCTGAACCCATGGTCAATTAACGAGGCAGTTTCTAGCTACTGCACGTA
CTTCATAAAGCAGGACTCTAAAAGCTTTGGAATCATGGTGTCATGGAAAG
GGATTTACTTTATACTGACTCTGTTTTGGGGAAGCTTTTTTGGAAGCATT
TTCATGCTGAGTCCCTTTTTACCTTTGATGTTTGTAAACCCATCTTGGTA
TCGCTGGATCAACAACCGCCTTGTGGCAACATGGCTCACCCTACCTGTGG
CATTATTGGAGACCATGTTTGGTGTAAAAGTGATTATAACTGGGGATGCA
TTTGTTCCTGGAGAAAGAAGTGTCATTATCATGAACCATCGGACAAGAAT
GGACTGGATGTTCCTGTGGAATTGCCTGATGCGATATAGCTACCTCAGAT
TGGAGAAAATTTGCCTCAAAGCGAGTCTCAAAGGTGTTCCTGGATTTGGT
TGGGCCATGCAGGCTGCTGCCTATATCTTCATTCATAGGAAATGGAAGGA
TGACAAGAGCCATTTCGAAGACATGATTGATTACTTTTGTGATATTCACG
AACCACTTCAACTCCTCATATTCCCAGAAGGGACTGATCTCACAGAAAAC
AGCAAGTCTCGAAGTAATGCATTTGCTGAAAAAAATGGACTTCAGAAATA
TGAATATGTTTTACATCCAAGAACTACAGGCTTTACTTTTGTGGTAGACC
GTCTAAGAGAAGGTAAGAACCTTGATGCTGTCCATGATATCACTGTGGCG
TATCCTCACAACATTCCTCAATCAGAGAAGCACCTCCTCCAAGGAGACTT
TCCCAGGGAAATCCACTTTCACGTCCACCGGTATCCAATAGACACCCTCC
CCACATCCAAGGAGGACCTTCAACTCTGGTGCCACAAACGGTGGGAAGAG
AAAGAAGAGAGGCTGCGTTCCTTCTATCAAGGGGAGAAGAATTTTTATTT
TACCGGACAGAGTGTCATTCCACCTTGCAAGTCTGAACTCAGGGTCCTTG
TGGTCAAATTGCTCTCTATACTGTATTGGACCCTGTTCAGCCCTGCAATG
TGCCTACTCATATATTTGTACAGTCTTGTTAAGTGGTATTTTATAATCAC

CATTGTAATCTTTGTGCTGCAAGAGAGAATATTTGGTGGACTGGAGATCA
TAGAACTTGCATGTTACCGACTTTTACACAAACAGCCACATTTAAATTCA
AAGAAAAATGAGCTCGAGATGGTGAGCAAGGGCGAGGAGGATAACATGGC
CATCATCAAGGAGTTCATGCGCTTCAAGGTGCACATGGAGGGCTCCGTGA
ACGGCCACGAGTTCGAGATCGAGGGCGAGGGCGAGGGCCGCCCCTACGAG
GGCACCCAGACCGCCAAGCTGAAGGTGACCAAGGGTGGCCCCCTGCCCTT
CGCCTGGGACATCCTGTCCCCTCAGTTCATGTACGGCTCCAAGGCCTACG
TGAAGCACCCCGCCGACATCCCCGACTACTTGAAGCTGTCCTTCCCCGAG
GGCTTCAAGTGGGAGCGCGTGATGAACTTCGAGGACGGCGGCGTGGTGAC
CGTGACCCAGGACTCCTCCCTGCAGGACGGCGAGTTCATCTACAAGGTGA
AGCTGCGCGGCACCAACTTCCCCTCCGACGGCCCCGTAATGCAGAAGAAG
ACCATGGGCTGGGAGGCCTCCTCCGAGCGGATGTACCCCGAGGACGGCGC
CCTGAAGGGCGAGATCAAGCAGAGGCTGAAGCTGAAGGACGGCGGCCACT
ACGACGCTGAGGTCAAGACCACCTACAAGGCCAAGAAGCCCGTGCAGCTG
CCCGGCGCCTACAACGTCAACATCAAGTTGGACATCACCTCCCACAACGA
GGACTACACCATCGTGGAACAGTACGAACGCGCCGAGGGCCGCCACTCCA
CCGGCGGCATGGACGAGCTGTACAAGGCGGCCGCATAGATAACTGATCCA
GTGTGCTGGAATTAATTCGCTGTCTGCGAGGGCCAGCTGT

TGGGGTGAGT

ACTCCCTCTCAAAAGCGGGCATGACTTCTGCGCTAAGATT

GTCAGTTTCC

AAAAACGAGGAGGATTTGATATTCACCTGGCCCGCGGTGA

TGCCTTTGAG

GGTGGCCGCGTCCATCTGGTCAGAAAAGACAATCTTTTTG

TTGTCAAGCT

TGAGGTGTGGCAGGCTTGAGATCTGGCCATACACTTGAGT

GACAATGACA

TCCACTTTGCCTTTCTCTCCACAGGTGTCCACTCCCAGGTCC

AACTGCAG

GTCGAGCATGCATCTAGGGCGGCCAATTCCGCCCCTCTCC

CTCCCCCCCC

CCTAACGTTACTGGCCGAAGCCGCTTGGAATAAGGCCGGT

GTGCGTTTGT

CTATATGTTATTTTCCACCATATTGCCGTCTTTTGGCAATGT

GAGGGCCC

GGAAACCTGGCCCTGTCTTCTTGACGAGCATTCCTAGGGG

TCTTTCCCCT

CTCGCCAAAGGAATGCAAGGTCTGTTGAATGTCGTGAAGG
AAGCAGTTCC

TCTGGAAGCTTCTTGAAGACAAACAACGTCTGTAGCGACC
CTTTGCAGGC

AGCGGAACCCCCCACCTGGCGACAGGTGCCTCTGCGGCCA
AAAGCCACGT

GTATAAGATACACCTGCAAAGGCGGCACAACCCCAGTGCC
ACGTTGTGAG

TTGGATAGTTGTGGAAAGAGTCAAATGGCTCTCCTCAAGC
GTATTCAACA

AGGGGCTGAAGGATGCCCAGAAGGTACCCCATTGTATGG
GATCTGATCTG

GGGCCTCGGTGCACATGCTTTACATGTGTTTAGTCGAGGT
TAAAAAAACG

TCTAGGCCCCCCGAACCACGGGGACGTGGTTTTCCTTTGA
AAAACACGAT

GATAAGCTTGCCACAACCCGGGATAATTCCTGCAGCCAAT
ATGGGATCGG

CCATTGAACAAGATGGATTGCACGCAGGTTCTCCGGCCGC
TTGGGTGGAG

AGGCTATTCGGCTATGACTGGGCACAACAGACAATCGGCT
GCTCTGATGC

CGCCGTGTTCCGGCTGTCAGCGCAGGGGCGCCCGGTTCTT
TTTGTCAAGA

CCGACCTGTCCGGTGCCCTGAATGAACTGCAGGACGAGGC
AGCGCGGCTA

TCGTGGCTGGCCACGACGGGCGTTCCTTGCGCAGCTGTGC
TCGACGTTGT

CACTGAAGCGGGAAGGGACTGGCTGCTATTGGGCGAAGT
GCCGGGGCAGG

ATCTCCTGTCATCTCACCTTGCTCCTGCCGAGAAAGTATCC

ATCATGGCT

GATGCAATGCGGCGGCTGCATACGCTTGATCCGGCTACCT

GCCCATTCGA

CCACCAAGCGAAACATCGCATCGAGCGAGCACGTACTCGG

ATGGAAGCCG

GTCTTGTCGATCAGGATGATCTGGACGAAGAGCATCAGG

GGCTCGCGCCA

GCCGAACTGTTCGCCAGGCTCAAGGCGCGCATGCCCGACG

GCGATGATCT

CGTCGTGACCCATGGCGATGCCTGCTTGCCGAATATCATG

GTGGAAAATG

GCCGCTTTTCTGGATTCATCGACTGTGGCCGGCTGGGTGT

GGCGGACCGC

TATCAGGACATAGCGTTGGCTACCCGTGATATTGCTGAAG

AGCTTGGCGG

CGAATGGGCTGACCGCTTCCTCGTGCTTTACGGTATCGCC

GCTCCCGATT

CGCAGCGCATCGCCTTCTATCGCCTTCTTGACGAGTTCTTC

TGAGGGGAT

CAATTCTCTAGATAACTGATCATAATCAGCCATACCACATT

TGTAGAGGT

TTTACTTGCTTTAAAAAACCTCCCACACCTCCCCCTGAACCT

GAAACATA

AAATGAATGCAATTGTTGTTGTTAACTTGTTTATTGCAGCT

TATAATGGT

TACAAATAAAGCAATAGCATCACAAATTTCACAAATAAAG

CATTTTTTTC

ACTGCATTCTAGTTGTGGTTTGTCCAAACTCATCAATGTAT

CTTAACGCG

TCGAGTGCATTCTAGTTGTGGTTTGTCCAAACTCATCAATG

TATCTTATC

ATGTCTGTATACCGTCGACCTCTAGCTAGAGCTTGGCGTAA

TCATGGTCA

TAGCTGTTTCCTGTGTGAAATTGTTATCCGCTCACAATTCC

ACACAACAT

ACGAGCCGGAAGCATAAAGTGTAAAGCCTGGGGTGCCTA

ATGAGTGAGCT

AACTCACATTAATTGCGTTGCGCTCACTGCCCGCTTTCCAG

TCGGGAAAC

CTGTCGTGCCAGCTGCATTAATGAATCGGCCAACGCGCGG

GGAGAGGCGG

TTTGCGTATTGGGCGCTCTTCCGCTTCCTCGCTCACTGACT

CGCTGCGCT

CGGTCGTTCGGCTGCGGCGAGCGGTATCAGCTCACTCAAA

GGCGGTAATA

CGGTTATCCACAGAATCAGGGGATAACGCAGGAAAGAAC

ATGTGAGCAAA

AGGCCAGCAAAAGGCCAGGAACCGTAAAAAGGCCGCGTT

GCTGGCGTTTT

TCCATAGGCTCCGCCCCCCTGACGAGCATCACAAAAATCG

ACGCTCAAGT

CAGAGGTGGCGAAACCCGACAGGACTATAAAGATACCAG

GCGTTTCCCCC

TGGAAGCTCCCTCGTGCGCTCTCCTGTTCCGACCCTGCCGC

TTACCGGAT

ACCTGTCCGCCTTTCTCCCTTCGGGAAGCGTGGCGCTTTCT

CAATGCTCA

CGCTGTAGGTATCTCAGTTCGGTGTAGGTCGTTCGCTCCAA

GCTGGGCTG

TGTGCACGAACCCCCCGTTCAGCCCGACCGCTGCGCCTTAT
CCGGTAACT

ATCGTCTTGAGTCCAACCCGGTAAGACACGACTTATCGCC
ACTGGCAGCA

GCCACTGGTAACAGGATTAGCAGAGCGAGGTATGTAGGC
GGTGCTACAGA

GTTCTTGAAGTGGTGGCCTAACTACGGCTACACTAGAAGG
ACAGTATTTG

GTATCTGCGCTCTGCTGAAGCCAGTTACCTTCGGAAAAAG
AGTTGGTAGC

TCTTGATCCGGCAAACAAACCACCGCTGGTAGCGGTGGTT
TTTTTGTTTG

CAAGCAGCAGATTACGCGCAGAAAAAAAGGATCTCAAGA
AGATCCTTTGA

TCTTTTCTACGGGGTCTGACGCTCAGTGGAACGAAAACTC
ACGTTAAGGG

ATTTTGGTCATGAGATTATCAAAAAGGATCTTCACCTAGAT
CCTTTTAAA

TTAAAAATGAAGTTTTAAATCAATCTAAAGTATATATGAGT
AAACTTGGT

CTGACAGTTACCAATGCTTAATCAGTGAGGCACCTATCTCA
GCGATCTGT

CTATTTCGTTCATCCATAGTTGCCTGACTCCCCGTCGTGTA
GATAACTAC

GATACGGGAGGGCTTACCATCTGGCCCCAGTGCTGCAATG
ATACCGCGAG

ACCCACGCTCACCGGCTCCAGATTTATCAGCAATAAACCAG
CCAGCCGGA

AGGGCCGAGCGCAGAAGTGGTCCTGCAACTTTATCCGCCT
CCATCCAGTC

TATTAATTGTTGCCGGGAAGCTAGAGTAAGTAGTTCGCCA

GTTAATAGTT

TGCGCAACGTTGTTGCCATTGCTACAGGCATCGTGGTGTC

ACGCTCGTCG

TTTGGTATGGCTTCATTCAGCTCCGGTTCCCAACGATCAAG

GCGAGTTAC

ATGATCCCCCATGTTGTGCAAAAAAGCGGTTAGCTCCTTCG

GTCCTCCGA

TCGTTGTCAGAAGTAAGTTGGCCGCAGTGTTATCACTCAT

GGTTATGGCA

GCACTGCATAATTCTCTTACTGTCATGCCATCCGTAAGATG

CTTTTCTGT

GACTGGTGAGTACTCAACCAAGTCATTCTGAGAATAGTGT

ATGCGGCGAC

CGAGTTGCTCTTGCCCGGCGTCAATACGGGATAATACCGC

GCCACATAGC

AGAACTTTAAAAGTGCTCATCATTGGAAAACGTTCTTCGG

GGCGAAAACT

CTCAAGGATCTTACCGCTGTTGAGATCCAGTTCGATGTAAC

CCACTCGTG

CACCCAACTGATCTTCAGCATCTTTTACTTTCACCAGCGTTT

CTGGGTGA

GCAAAAACAGGAAGGCAAAATGCCGCAAAAAAGGGAATA

AGGGCGACACG

GAAATGTTGAATACTCATACTCTTCCTTTTTCAATATTATTG

AAGCATTT

ATCAGGGTTATTGTCTCATGAGCGGATACATATTTGAATGT

ATTTAGAAA

AATAAACAAATAGGGGTTCCGCGCACATTTCCCCGAAAAG

TGCCACCTGA

CGTC
